# Analytical and Functional Similarity Assessment of ABP 710, a Biosimilar to Infliximab Reference Product

**DOI:** 10.1007/s11095-020-02816-w

**Published:** 2020-05-31

**Authors:** Ramsey Saleem, Greg Cantin, Mats Wikström, Glen Bolton, Scott Kuhns, Helen J. McBride, Jennifer Liu

**Affiliations:** Amgen Inc., Thousand Oaks, California USA

**Keywords:** ABP 710, infliximab, biological function, biosimilar, physicochemical structure

## Abstract

**Purpose:**

ABP 710 has been developed as a biosimilar to infliximab reference product (RP). The objective of this study was to assess analytical similarity (structural and functional) between ABP 710 and infliximab RP licensed by the United States Food and Drug Administration (infliximab [US]) and the European Union (infliximab [EU]), using sensitive, state-of-the-art analytical methods capable of detecting minor differences in product quality attributes.

**Methods:**

Comprehensive analytical characterization utilizing orthogonal techniques was performed with 14 to 28 unique lots of ABP 710 or infliximab RP, depending on the assay. Comparisons were used to investigate the primary structure related to amino acid sequence; post-translational modifications (PTMs) including glycans; higher order structure; particles and aggregates; primary biological properties mediated by target and receptor binding; product-related substances and impurities; and general properties.

**Results:**

ABP 710 had the same amino acid sequence, primary structure, higher order structure, PTM profiles and biological activities as infliximab RP. The finished drug product had the same strength (protein content and concentration) as infliximab RP.

**Conclusions:**

Based on the comprehensive analytical similarity assessment, ABP 710 was found to be highly analytically similar to infliximab RP for all biological activities relevant for clinical efficacy and safety.

**Electronic supplementary material:**

The online version of this article (10.1007/s11095-020-02816-w) contains supplementary material, which is available to authorized users.

## Introduction

ABP 710 has been developed as a biosimilar to infliximab reference product (RP) (Remicade^®^). Remicade^®^ has been approved in over 30 countries including the United States (US), European Union (EU), Japan and China for adult and pediatric Crohn’s disease, adult and pediatric ulcerative colitis, rheumatoid arthritis, ankylosing spondylitis, psoriatic arthritis and plaque psoriasis (1). Infliximab RP is a chimeric immunoglobulin G (IgG) monoclonal antibody (mAb) produced in murine myeloma cells by recombinant DNA technology. The primary mechanism of action of infliximab RP is to neutralize the biological activity of tumor necrosis factor-alpha (TNFα) by binding with high affinity to the soluble TNFα (sTNFα) and membrane bound (mb) forms of TNFα (mbTNFα) and inhibit binding of TNFα with its receptors (2,3). Sequestration of sTNFα from interaction with TNFα receptors TNFR-1/p55 and TNFR-2/p75, and sequestration of mbTNFα from interaction with TNFR-2 mitigates pro-inflammatory signaling. The antigen binding fragment (Fab) of infliximab RP mediates the binding to TNFα and its subsequent neutralization; it can also induce apoptosis through reverse signaling in mbTNFα expressing cells. In addition, the region containing the crystallizable fragment (Fc) of the molecule mediates downstream effector functions. *In vitro*, infliximab RP can induce antibody-dependent cell-mediated cytotoxicity (ADCC) and complement-dependent cytotoxicity (CDC) of mbTNFα-expressing cells through the Fc-containing portion of the molecule (4).

A biosimilar is a biologic drug that is highly similar to an approved, branded biological RP (5,6). In order to improve access to biologic drugs, US Food and Drug Administration (FDA) and European Medicines Agency (EMA) guidelines were established to provide an abbreviated development and approval process for biosimilars. These guidelines require a stepwise approach to demonstrate similarity, which begins with the demonstration of analytical similarity (structural and functional) characteristics. Once this foundational similarity has been demonstrated, assessments of similarity of other characteristics (including non-clinical similarity that may comprise mechanism of action, pharmacokinetics (PK)/pharmacodynamics, and toxicokinetic studies) are performed. This is then followed by comparative clinical studies, first to evaluate PK and pharmacodynamics, if relevant. Finally, at least one comparative clinical study is conducted to evaluate the similarity of efficacy, safety and immunogenicity in at least 1 representative indication using a population and endpoints that are appropriately sensitive to complete the totality-of-evidence needed for approval. Phase 2 studies are not required during biosimilar development.

Assessment of analytical similarity begins with the identification of product quality attributes (PQAs) which can potentially impact safety, efficacy, PK and overall quality (7). The PQAs can be influenced by the host cell line that is selected for product expression. Infliximab RP was developed and manufactured using an SP2/0 murine cell line, whereas ABP 710 was developed and manufactured using a Chinese hamster ovary (CHO) cell line. SP2/0 cells generate mAbs that present with glycoforms with terminal galactose-alpha-1,3-galactose (α-Gal) and N-glycolylneuraminic acid (NGNA) (8,9). While these non-human glycans are present at relatively low levels in infliximab RP, a large proportion of the human population has been reported to have pre-existing antibodies with reactivity against α-Gal and NGNA (10,11). These pre-existing antibodies can theoretically bind to therapeutic mAbs containing the α-Gal and NGNA glycans, impacting safety, PK, and efficacy (12). For example, the presence of the α-Gal moiety in the glycans of cetuximab can lead to Ig isotype E (IgE)-mediated hypersensitivity reactions upon cetuximab administration in a subset of patients (13). Since the approval of infliximab (US) in 1998, CHO cell lines have been more commonly used than SP2/0 cell lines for the production of recombinant biological products and present a number of advantages, including potentially improved viral safety, reduced immunogenicity concerns and the ability to use biomanufacturing processes devoid of animal-derived materials. CHO cell lines are regarded as the current state-of-the-art mammalian expression system for the production of biopharmaceuticals due to high proliferation and high antibody production rates, as well as resistance to mechanical stress during manufacturing while maintaining proper glycosylation and folding (14,15).

Considering these factors, CHO cell lines were selected as the expression host for ABP 710. Since CHO expressed mAbs are known to express N-acetylneuraminic acid (NANA) sialylation as opposed to NGNA, and are effectively free of terminal α-Gal glycans, the potential for immunogenicity or safety concerns in the subset of patients with pre-existing antibodies is minimized. Given that a change in expression system may produce a slightly different glycan profile as compared to the RP, a carefully considered clone selection process was applied with an emphasis on maintaining biological function and minimizing any potential impacts on safety and efficacy (16). The PQAs, including glycosylation profile and biologically relevant functions, were characterized and monitored during development to aid in the final cell line and clone selection. Although cell line selection is driven by the ability of a given cell line to produce proteins that are highly similar in structure and biological functions to the RP, many factors related to large-scale production, including cell line expansion, bioreactor conditions, protein purification, formulation and packaging, must also be carefully evaluated due to potential impact on PQA.

Here we describe the methods and results of the comprehensive analysis of the structural and functional similarity of ABP 710 to infliximab RP. This comprehensive analytical similarity characterization was designed to assess biochemical, biophysical and biological similarity to ensure the understanding of whether any differences between ABP 710 and infliximab RP have the potential to impact clinical performance, consistent with US and EU guidelines (6,17). In these studies, infliximab RP was sourced from both US (infliximab US) and EU (infliximab EU). This was done to satisfy the regulatory requirement, which states that the RP in these foundational comparative assessments must be locally sourced. This allows for the development of a scientific bridge such that only a single source of RP could be used in clinical evaluations.

The first approval of a biosimilar monoclonal antibody in the US and EU was a biosimilar to infliximab RP (Inflectra™) (18). The methods and results of similarity assessments of previously approved biosimilars to infliximab RP are available and provide a rich dataset around the impact of cell line and manufacturing process on PQAs and the relationship to biological activities (19–25). This work showing the analytical similarity assessment for ABP 710, a molecule designed to match biological functions even in light of well understood analytical differences due to the difference in cell line, adds to our scientific understanding of the development of a biosimilar product and informs the approaches for design and control of novel monoclonal antibodies for human therapy.

## Materials and Methods

Several primary and orthogonal assays were performed to assess the similarity of ABP 710 with the RP. ABP 710 was manufactured by Amgen Inc. US-licensed infliximab, manufactured by Janssen Biotech, Inc. and EU-authorized infliximab, manufactured by Janssen Biologics B.V. was sourced over a period of approximately 6 years. These infliximab RPs were stored and handled according to the manufacturer’s instructions and tested as part of the analytical similarity assessment plan. ABP 710 has the same formulation as infliximab RP and was developed to reflect the same strength and presentations approved for infliximab in the US and the EU (1).

### Intact Molecular and Reduced Deglycosylated Molecular Mass Analysis

The molecular masses of intact molecules were determined by electrospray ionization-time of flight-mass spectrometer analysis. Samples were separated from buffer components and introduced to a time-of-flight (TOF) MS by electrospray ionization (ESI) after separation by SE chromatography using a BEH 200 SEC (4.6 × 150 mm), 1.7 μm Ethylene Bridged Hybrid column (Waters, Milford, MA). The resulting summed ion spectra were deconvoluted to produce molecular mass profiles. For deglycosylated analyses, samples were treated with PNGase F (NEB) to remove N-linked glycans. Reduced samples were treated with dithiothreitol (DTT, Thermo Scientific) to reduce the disulfides.

### Reduced Peptide Map

Reduced peptide map analysis was conducted by enzymatic digestion with trypsin (Roche Life Science). The sample was denatured with 6.5 M guanidine, 100 mM Tris pH 8.3, then reduced with 10 mM DTT and alkylated using 20 mM sodium iodoacetic acid (IAA, Sigma-Aldrich). N-linked glycans were removed using PNGase F for the N-linked glycan, and the deglycoslated material desalted was then digested with a 10:1 ratio of protein:trypsin. After quenching with trifluoracetic acid (TFA), the resulting peptides were separated on a C18 reversed phase column using ultra-high performance liquid chromatography (UHPLC) with an increasing gradient of acetonitrile in water (from 99% acetonitrile 0.1% TFA to 45% acetonitrile 0.1% TFA over 123 min). Eluting peaks were detected by ultraviolet (UV) absorbance (214 nm), and the peptides were introduced to the mass spectrometer by ESI. Peptide masses were determined by on-line MS (LC-MS/MS) using a hybrid Ion Trap-Orbitrap Mass Spectrometer.

### Glycan Map

N-linked glycans (N-glycans) were evaluated by glycan map analysis using hydrophilic interaction liquid chromatography (HILIC) with fluorescence detection. N-glycans were released by treatment with PNGase F. The reducing termini of the released glycans were then labeled through reductive amination with a fluorescent tag, 2 aminobenzoic acid (2-AA, Sigma Aldrich). Following incubation at 80°C, precipitated proteins were pelleted and the supernatant, containing released and labeled glycans, was transferred to a new vial. The labeled glycans were separated by HILIC-UHPLC on a BEH Glycan column (2.1 × 150 mm, 1.7 μm; Waters Corporation), using a 100 mM ammonium formate, pH 3.0 and acetonitrile gradient. The gradient was 22% 100 mM ammonium formate to 40% over 111 min. Peak identification was performed using MS by coupling the HILIC UHPLC with an ion-trap MS for verification against the expected glycan mass.

### Fourier-Transform Infrared (FTIR) and Near-UV Circular Dichroism (NUV-CD)

The secondary structure was assessed by Fourier-transform infrared spectroscopy (FTIR) and recorded at ambient temperature using a Bruker Vertex 70 spectrometer. The samples were measured directly in the Aqua Spec Cell that employs CaF2 windows separated by a 6-µm spacer. For each spectrum, a 256-scan interferogram was collected in a single beam mode, with a 4 cm^−1^ resolution. The spectrum of the formulation buffer blank was recorded under identical conditions as the protein spectrum and subtracted from the protein solution spectra. The second derivative spectrum was calculated using a 9-point smoothing of the resulting spectrum using the Bruker OPUS software. The spectral similarity of the second-derivative FTIR spectra in the amide I region (1700–1600 cm^−1^) was calculated by comparing the second-derivative spectrum of the testing molecule against the reference molecule using the Thermo OMNIC software QC Compare function where 100% similarity indicates identical spectra (26). The tertiary structure was assessed by near-UV circular dichroism (NUV-CD) spectroscopy. The NUV-CD measurements were made on an Applied Photophysics Chirascan spectropolarimeter (Applied Photophysics Ltd., Leatherhead, UK) at ambient temperature. The protein solutions were diluted to approximately 0.7 mg/mL in formulation buffer, and the NUV-CD measurements were performed using cuvettes with a path length of 1 cm. The spectra were recorded using a step size of 0.5 nm, a bandwidth of 1 nm, a response time of 2 s, a 4-scan average, with the wavelength range from 340 to 240 nm. The spectra were corrected for protein concentration and contributions from the buffer and were reported as CD ellipticity. Spectral similarity was calculated using the Thermo OMNIC software QC Compare function where 100% similarity indicates identical spectra (27).

### Differential Scanning Calorimetry (DSC)

Thermal stability was assessed by differential scanning calorimetry (DSC) using a MicroCal VP-capillary differential scanning calorimeter (Microcal/Malvern Instruments, Worcestershire, United Kingdom) in which temperature differences between the testing sample and buffer cell were continuously measured and calibrated. Thermal stability was assessed by DSC measurements that were made in 6 replicates with scans completed from 10°C to 100°C using a scan rate of 60°C/h. Post-run data analysis was used to determine the thermal transition temperature (Tm). Data analysis was performed using MicroCal Origin software, and the statistical analysis was performed using the JMP software. The protein solutions were diluted to approximately 0.5 mg/mL in formulation buffer.

### High Accuracy Light Obscuration (HIAC) and Micro-Flow Imaging (MFI)

Subvisible particles were assessed by light obscuration using a HIAC 9703C liquid particle counting system equipped with an HRLD 150 sensor. Particle concentration results were reported as cumulative particle counts per mL for ≥2, ≥ 5, ≥ 10 and ≥ 25 μm size ranges. Subvisible particles were also assessed by micro-flow imaging (MFI) particle imaging system containing a flow cell and a digital camera. Cumulative particle counts per mL for ≥5 μm particles were reported. To quantify product-related particles that are likely proteinaceous and thus have a higher risk for immunogenicity, the MFI data were further analyzed for the concentration of ≥5 μm non-spherical particles with an aspect ratio of <0.85.

### Sedimentation Velocity Analytical Ultracentrifugation (SV-AUC)

The SV-AUC analysis was performed using a Beckman Coulter ProteomeLab XL-I instrument. The sedimentation velocity experiments were performed at 45,000 rpm, and the absorbance at 280 nm were recorded. Experiments were performed in double-sector centerpiece cell assemblies with quartz windows. Scans were collected at 20°C without delay between them. The SV-AUC data were analyzed using SEDFIT (28). In the analysis, the frictional ratio, time invariant noise, and meniscus values were allowed to float during the nonlinear least squares fit. Product solutions were diluted with formulation buffer to approximately 0.5 mg/mL, and triplicate measurements were taken for each test solution.

### Size Exclusion HPLC (SE-HPLC) with LS Detection (SE-HPLC-LS)

The SE-HPLC-LS analysis was performed using an Agilent 1100 HPLC system with a TSK-GEL G3000SWxl, 5 μm particle size, 7.8 mm ID × 300 mm length column (Tosoh Biosep, 08541). The detectors used were a Wyatt HELEOS MALS detector, a Wyatt Optilab TrEX RI detector, and an Agilent UV detector with wavelength set at 280 nm. The SE-HPLC-LS runs were performed at room temperature with 100 mM sodium phosphate, 250 mM sodium chloride, pH 6.8 buffer used as the mobile phase, and the flow rate was 0.5 mL/min. Samples were injected neat into the SE-HPLC-LS system for a load of approximately 280 μg. For molar mass calculation, refractive index increment, dn/dc = 0.185 (mL/g), was used. Results were reported as the molar mass of monomer and high molecular weight (HMW) species using the Astra software (Wyatt Technologies Inc.).

### SE Ultra HPLC (SE-UHPLC)

Native size variants were analyzed by SE-UHPLC. SE-UHPLC measurements were made on a Waters Acquity UPLC with a Waters BEH200 1.7 μm, 4.6 × 150 mm SEC column. Samples were run isocratically using 250 mM sodium chloride, 100 mM sodium phosphate, 10% acetonitrile, pH 6.8. Analytes were monitored by UV absorbance at 280 nm, and purity was evaluated by determining the peak area of each species as a percentage of the total peak area.

### Reduced and Non-reduced Capillary Electrophoresis-Sodium Dodecyl Sulfate (CE-SDS)

Capillary electrophoresis–sodium dodecyl sulfate (CE-SDS) was used for separation of denatured protein size variants under reduced or non-reduced conditions. For non-reduced condition, drug product samples were denatured using 1.3% SDS and 5 mM N-ethylmalimide (NEM) at 60°C for 5 min. For reduced condition, β-mercaptoethanol was added to the protein denaturation step to reduce the disulfide bonds and incubation was performed at 70°C for 10 min. After denaturation, samples were injected onto a bare, fused silica capillary and separated based on hydrodynamic size resulting from an applied electric field in which migration time of smaller size proteins is inversely related to overall size. Analytes were monitored by UV absorbance at 220 nm, and purity was evaluated by determining the peak area of each species as a percentage of the total peak area.

### Cation Exchange-High Performance Liquid Chromatography (CEX-HPLC)

Charged isoforms in drug product samples were separated on a CEX-HPLC Proteomix SCX cation, 5 μm, 4.6 × 100 mm analytical column. A gradient of 20 mM sodium phosphate, pH 6.5, and 20 mM sodium phosphate, 500 mM sodium chloride, pH 6.5, going from 100% to 90% 20 mM sodium phosphate, pH 6.5, over 30 min, was used. Eluting species were monitored by UV absorbance at 280 nm and purity was evaluated by determining the peak area of each charged isoform group (acidic, main, and basic peaks) that eluted separately as a percentage of the total peak area.

### Protein Concentration and Protein Content

The protein concentration in the solution and protein content in the vial was determined by UV absorbance using the product extinction coefficient.

### sTNFα Binding

sTNFα was coated onto the wells of microtiter enzyme-linked immunosorbent assay (ELISA) plates. After blocking, a serial dilution of reference standard, control and test sample(s) was added. Following a wash step, a goat anti-human IgG Fc secondary antibody, conjugated with horseradish peroxidase was added to detect bound samples. After a final wash, the chromogenic horseradish peroxidase substrate was added to the wells. The reaction was stopped with 1.0 M phosphoric acid and absorbance was measured with a microplate reader. Dose-response data were fit to a 4-parameter curve, and the half maximal effective concentration (EC50) values derived from the curves were used to calculate the test sample relative binding activity.

### mbTNFα Binding

Binding of ABP 710 and infliximab RP to mbTNFα was evaluated in a cell-based competitive binding assay using CHO MT-3 cells expressing a non-cleavable form of human mbTNFα (4). In this method, the test sample competes with a fixed concentration of ABP 710 labeled with Alexa488 for binding to the mbTNFα expressed on the engineered CHO cells. The dose-dependent decrease in cell-bound fluorescence was measured by an image cytometer. Dose-response data were fit to a 4-parameter curve, and the half maximal inhibitor concentration (IC50) values derived from the curves were used to calculate the test sample relative binding activity.

### Inhibition of sTNFα-Induced Apoptosis (Potency)

The ability of ABP 710 to inhibit sTNFα-induced apoptosis was evaluated using the human histiocytic lymphoma cell line U-937. sTNFα induces U-937 cells to undergo apoptosis through TNFR1 signal-mediated caspase activation. A luminogenic substrate containing the DEVD amino acid sequence (Asp-Glu-Val-Asp) was recognized and cleaved by the activated caspases. Cleavage of the DEVD sequence from the luminogenic substrate results in light (luminescence) production that is detected by a Caspase-Glo^®^ 3/7 Assay System kit. The amount of luminescence generated is proportional to the amount of caspase activation and is quantified in a luminometer. Reference standard, control and test sample(s) cause a dose-dependent inhibition of TNFα-induced caspase activation, in turn leading to a reduced level of apoptosis. Dose-response data were fit to a 4-parameter curve, and the EC50 values derived from the curves were used to calculate the test sample relative activity.

### NK92 Antibody-Dependent Cell-Mediated Cytotoxicity (ADCC) Assay

The NK92 ADCC activity of ABP 710 was evaluated in a cell-based assay using CHO MT-3 cells expressing a non-cleavable form of human mbTNFα. NK92-M1 cells, stably transfected with human CD16 (FcγRIIIa-158V) were used as effector cells. NK92-M1/CD16 effector cells and calcein-acetoxymethyl (AM) loaded target cells were incubated at a 15:1 effector to target ratio with increasing concentrations (0.01 ng/mL–100,000 ng/mL) of reference standard, control and test sample(s). Upon ADCC-mediated target cell lysis, calcein is released from the target cells into the cell culture medium and is measured with a fluorimeter. The amount of culture supernatant fluorescence detected is proportional to the amount of ADCC activity. Dose-response data were fit to a 4-parameter curve, and the EC50 values derived from the curves were used to calculate the test sample relative ADCC activity.

### Peripheral Blood Mononuclear Cell (PBMC) ADCC Assay

PBMCs isolated from healthy donors, heterozygous for FcγRIIIa-158(V/F) are used as effector (E) cells. CHO cells constitutively expressing both a non-cleavable version of mbTNFα and a luciferase reporter gene are used as target (T) cells. Using previously determined donor-specific E:T ratios that would provide a suitable level of target death for the purposes of conducting a similarity assessment, T and E cells are incubated with increasing concentrations of test samples for 20 to 22 h. After incubation, Steady-Glo^®^ Luciferase Assay Reagent is added to the assay plates to measure the presence of intracellular luciferase activity that is released from membrane-compromised cells. The quantity of dead-cell luciferase in the medium is directly proportional to the number of dead cells in the culture. Dose-response data were fit to a 4-parameter curve, and the EC50 values derived from the curves were used to calculate the test sample relative activity.

### Complement-Dependent Cytotoxicity (CDC) Assay

The CDC activity of ABP 710 was evaluated in a functional cell-based assay. Target CHO MT-3 cells expressing mbTNFα were loaded with the fluorescent dye, calcein-AM. The calcein-AM loaded target cells were incubated with different dose concentrations of reference standard, control and test sample(s) followed by addition of rabbit complement. After allowing for complement-mediated cell lysis to take place, the amount of cellular lysis induced by the antibody was measured using a fluorimeter. Dose-response data were fit to a 4-parameter curve, and the EC50 values derived from the curves were used to calculate the test sample relative activity.

### Reverse Signaling Assay

An engineered Jurkat cell line constitutively expressing a non-cleavable version of mbTNFα was used to assess the induction of apoptosis through direct activation of an mbTNFα-specific reverse signaling pathway in the presence of ABP 710. Upon the early stages of apoptosis, the intracellular cell membrane surface phospholipid phosphatidylserine molecules are transferred to the outer surface of the cell membrane. Fluorochrome-labeled Annexin V (Annexin-FITC) binds to phosphatidylserine and allows for detection of apoptotic cells via flow cytometry. Inclusion of propidium iodide stain, which is unable to enter cells with intact cellular membranes, allows for differentiation of membrane compromised dead cells (propidium iodide positive). When reference standard, control and test sample(s) are present, a dose-dependent increase in Annexin-FITC labeled cells is observed. Dose-response data were fit to a 4-parameter curve, and the EC50 values derived from the curves were used to calculate the test sample relative activity.

### Antibody-Dependent Cellular Phagocytosis (ADCP) Assay

CHO cells expressing non-cleavable human mbTNFα were fluorescently labelled with eFluor670 for 10 min before dilution in growth medium. Labelled CHO cells were pelleted by centrifugation, suspended in fluorescence-activated cell sorting buffer, and combined with PBMCs isolated from healthy donors in a 1:5 ratio. Titrated dilutions of reference standard, control and test sample(s) were added to each well. The cells were incubated with test sample and subsequently stained with anti-CD14 antibody to stain macrophages. Cells were gated on the CD14 positive cell population. The cell population considered positive for ADCP activity was assessed based on cells that stained for both CD14 and the target cell label. Data analysis was performed using FlowJo software. Dose-response data were fit to a 4-parameter curve, and the EC50 values derived from the curves were used to calculate the test sample relative activity.

### Neonatal Fc receptor (FcRn) Binding

A competitive AlphaScreen^®^ binding assay was used to assess the binding of ABP 710 FcRn. AlphaScreen^®^ is a bead-based, amplified luminescent proximity homogenous assay. The Fc region from reference standard, control and test sample(s) and a fixed concentration of Fc-biotin in solution compete for binding to histidine-tagged FcRn (FcRn-his). FcRn-his captured on the acceptor beads binds to the Fc-biotin captured on the donor beads, resulting in luminescence. Reference standard, control and test sample(s) compete for binding. Dose-response data were fit to a 4-parameter curve, and the IC50 values derived from the curves were used to calculate the test sample relative activity.

### FcRn Binding by Surface Plasmon Resonance (SPR)

A surface plasmon resonance (SPR) assay analogous to the FcγRIIa (131R) binding assay was developed, except that the CM5 chip was coated with recombinant human FcRn. FcRn was immobilized through standard amine coupling chemistry to a Biacore CM5 chip. Binding data were collected 4 seconds prior to the end of injection for each concentration of a duplicate sample dilution series. The Kd was determined using the steady state affinity model. The binding data generated from the assay run was used to determine the relative binding activity of the test samples in comparison to the reference standard by dividing the Kd of the sample by that of the reference standard.

### Binding to Lymphotoxin Alpha (LTα)

Binding to LTα was assessed by SPR using a Biacore T200 instrument. ABP 710, infliximab (US), infliximab (EU), or Amgen-manufactured etanercept (positive control) were captured on a CM5 sensor chip surface using a goat-anti-human IgG1 antibody. A single saturating concentration of recombinant human LTα (30 nM) was injected for 90 s followed by a dissociation time of 20 s. Immediately afterward, a saturating concentration of recombinant human sTNFα (30 nM) was injected for 90 s followed by a dissociation time of 20 s. Each test sample was analyzed in duplicate. Capture and binding responses were assessed using Biacore software.

### Inhibition of LTα-Induced Interleukin 8 (IL-8) Release

Release of IL-8 in human umbilical vein cells (HUVEC) in the presence of LTα was measured using a human IL-8 homogeneous time resolved fluorescence (HTRF^®^) assay. Reference standard, control and test sample(s) dilutions were pre-incubated with LTα before transferring the antibody/LTα mixture to the cells. Cells were incubated, and an IL-8 detection reagent was added that consists of 2 non-competing anti-IL-8 antibodies labeled with a donor molecule (europium cryptate) on 1 of the antibodies and an acceptor molecule (allophycocyanin or XL665) on the other. Plates are read using an HTRF^®^ compatible plate reader. Dose-response data were fit to a 4-parameter curve, and the IC50 values derived from the curves were used to calculate the test sample relative activity. A mouse IgG1 monoclonal anti-human LTα antibody was included in each assay run as a positive control. The basal % IL-8 release control for HUVEC alone and the % IL-8 release for HUVEC in the presence of LTα are shown plotted on the same graph for comparison.

### Soluble Tumor Necrosis Factor Alpha (sTNFα) Binding

Binding to sTNFα was assessed by SPR using a Biacore T200 instrument. Anti-human IgG (Fc) antibody was immobilized on a CM5 sensor chip surface. Reference standard, control or test sample(s) were captured on the chip surface through binding to anti-human IgG (Fc). Recombinant human sTNFα was used as the analyte. The data were aligned and fit using the Biacore T200 Evaluation software. The dissociation equilibrium binding constant (Kd) was determined using the 1:1 Langmuir binding model, and the percent relative binding of each sample to the reference standard was determined.

### sTNFα Binding Kinetics

Binding to sTNFα was assessed by SPR using a Biacore T200 instrument. Reference standard, control or test sample(s) were captured on a CM5 sensor chip surface using a goat-anti-human IgG1 antibody. Recombinant human sTNFα was used as the analyte. Results are reported as the average of 3 intra-assay replicates per sample. The data were aligned and fit using an SPR data processing and non-linear least squares regression fitting program. First, a dissociation rate coefficient (kd) was determined by globally fitting the 600-s dissociation phase data to a simple exponential decay model. Second, this value was applied as a fixed parameter in the global fit of the 240-s association phase data to a 1:1 binding model to determine the association rate coefficient (ka) and the dissociation equilibrium binding constant (Kd).

### Inhibition of sTNFa-Induced Cell Death

L-929 cells were plated and allowed time for attachment before exposure to actinomycin D to sensitize the cells to sTNFα. Reference standard, control or test sample(s) dilutions were pre-incubated with the cells after sensitization. sTNFα was then added to each well, and plates were placed in an incubator. After 24 h, the medium was removed, and the remaining cells were lysed and quantified using an assay kit that measures lysed cells with a luminescent substrate. Dose-response data were fit to a 4-parameter curve, and the IC50 values derived from the curves were used to calculate the test sample relative activity.

### FcγRIa Binding

Relative binding to FcγRIa was quantified using AlphaLISA^®^ assays (Perkin Elmer). FcγRIa GST-fusion proteins were generated at Amgen, Inc. Thousand Oaks, CA, USA. The AlphaLISA^®^ assay donor beads were coated with a hydrogel that contains phthalocyanine, a photosensitizer and streptavidin, which binds to biotinylated human IgG1 (Amgen). When reference standard, control or test sample(s) are present they inhibit the binding of FcγR-GST to the biotinylated human IgG1. Dose-response data were fit to a 4-parameter curve, and the IC50 values derived from the curves were used to calculate the test sample relative activity.

### FcγRIIa (131R), FcγRIIb, FcγRIIIa (158V), FcγRIIIa (158F) and FcγRIIIb Binding

FcγRIIa (131R), FcγRIIb, FcγRIIIa (158V), FcγRIIIa (158F) or FcγRIIIb (R&D Systems) was immobilized through standard amine coupling chemistry to a BiaCore CM5 chip (GE Healthcare Bio-Sciences). The Kd was determined using the steady state affinity model. The binding data generated from the assay run was used to determine the relative binding activity of the test samples in comparison to the reference standard by dividing the Kd of the sample by that of the reference standard.

### FcγRIIIa (158V) Binding Kinetics

Binding to FcγRIIIa (158V) was assessed by SPR using a Biacore T200 instrument. Recombinant human TNFα was immobilized on a CM5 sensor chip surface. Reference standard, control or test sample(s) were captured on the chip surface through binding to recombinant human TNFα. Results are reported as the average of 3 intra-assay replicates per lot. The data were aligned and fit using an SPR data processing and non-linear least squares regression fitting program. The association and dissociation phase data were simultaneously, globally fit to a 1:1 binding model to determine the association rate coefficient (ka), the dissociation rate coefficient (kd), and the dissociation equilibrium binding constant (Kd).

### First Sub-Component of Complement (C1q) Binding

A direct binding ELISA method was developed to assess the binding of reference standard, control or test sample(s) to C1q (Sigma-Aldrich). Reference standard, control or test sample(s) was adsorbed to a microtiter plate and incubated with C1q. Bound C1q was detected with an anti-C1q-horseradish peroxidase conjugated antibody (Bio-Rad). After a final wash, a substrate/chromogen solution was added to the wells.

The reaction was stopped with sulfuric acid and absorbance was measured with a microplate reader. Dose-response data were fit to a 4-parameter curve, and the IC50 values derived from the curves were used to calculate the test sample relative activity.

## Results

The testing plan listing all the analytical techniques and attributes evaluated for ABP 710 and infliximab RP is shown in Table I. The objective was to evaluate both active ingredients and inactive ingredients that could affect product safety and efficacy in addition to product quality. Where applicable, orthogonal methods were used to fully analyze product attributes and activities. The ABP 710 batches used in the similarity assessment represent drug product lots filled from 14 individual drug substance lots manufactured over the same period. For parameters primarily influenced by the drug product manufacturing process (e.g., protein content), 19 drug product lots were included in the analytical similarity assessment, including all lots used in clinical studies and drug product process validation lots. A total of 28 infliximab (US) lots and 20 infliximab (EU) lots, including all lots used in the clinical trials, were sourced over a period of approximately 6 years and included in the analytical similarity assessment. All ABP 710 and infliximab RP lots were tested prior to the specified expiration date, using methods with suitable intermediate precision, to allow quantitative results to be generated as lots were manufactured or procured over time. For profile comparison (e.g., chromatogram overlays), ABP 710 and infliximab lots were tested prior to the specified expiration date and in a single experiment. Analytical similarity assessment was performed on lyophilized drug products wherever possible. The number of lots tested for each assay was determined by the knowledge of the product attributes and likelihood to be influenced by manufacturing processes (i.e., variability between lots). This vast dataset allows a meaningful and comprehensive assessment of comparisons that increases confidence in the overall conclusion of analytical similarity.

**Table I:** Similarity Testing Plan and the Analytical Methods for the Structural and Functional Characterization of the Proposed Biosimilar ABP 710 and Infliximab Reference Products

**Category**	**Attributes and Analytical Techniques**
General properties	Protein content by gravimetric analysis
	Reconstituted protein concentration by UV absorbance
	Reconstitution time
Primary structure and post-translational modifications	Intact molecular mass analysis
	Reduced and deglycosylated molecular masses of HC and LC
	Protein sequence by reduced peptide map
	Reduced peptide map, post-translational modifications: levels of deamidation and oxidation
	Disulfide structure by nonreduced peptide map
	Glycan map by HILIC
	Isoelectric point by capillary isoelectric focusing
	Identity by anti-idiotype ELISA
Higher order structure	Secondary structure by FTIR spectroscopy
	Tertiary structure by NUV-CD spectroscopy
	Conformation and thermal stability by DSC
Product-related substances and impurities	Size variants by SE-UHPLC, rCE-SDS, and nrCE-SDS
	Charge variants by CEX-HPLC and CEX-HPLC after carboxypeptidase B treatment
Particles and aggregates	Subvisible particles by HIAC and MFI
	Submicron particles by FFF and DLS
	Solution state size distribution by SV-AUC
	Molar mass of size variants by SE-HPLC-LS
Biological activity	Inhibition of sTNFα-induced apoptosis in U937
	Binding to sTNFα by ELISA
	Binding to sTNFα by SPR
	Binding kinetics to sTNFα by SPR
	Inhibition of sTNFα-induced IL-8 release in HUVEC
	Inhibition of sTNFα-induced cell death in L929
	Binding to mbTNFα on CHO MT-3 cells by imaging cytometry
	Reverse signaling in Jurkat mbTNFα line
	Binding to LTα by SPR
	Inhibition of LTα-induced IL-8 release in HUVEC
	Binding to FcγRIIIa (158V) by SPR
	Binding kinetics to FcγRIIIa (158V) by SPR
	Binding to FcγRIIIa (158F) by SPR
	Binding to primary NK cells by FACS
	NK92 ADCC activity
	PBMC ADCC activity
	Binding to C1q by ELISA
	CDC activity
	Binding to FcγRIIa (131R) by SPR
	ADCP activity
	Binding to FcγRIa by AlphaLISA
	Binding to FcγRIIb by SPR
	Binding to FcγRIIIb by SPR
	Binding to FcRn by AlphaScreen
	Binding to FcRn by SPR
Thermal stability and forced degradation	Reconstituted forced degradation study at 40°C
	Lyophilized stressed degradation study at 40°C
	Lyophilized accelerated degradation study at 25°C assessedby purity and potency assays

*ADCC* antibody-dependent cell-mediated cytotoxicity, *ADCP* antibody-dependent cellular phagocytosis, *SV-AUC* sedimentation velocity analytical ultracentrifugation, *CDC* complement-dependent cytotoxicity, *CEX-HPLC* cation exchange high performance liquid chromatography, *CHO* Chinese Hamster Ovary cell, *cIEF*,capillary isoelectric focusing, *C1q* the first subcomponent of the C1 complex of the classical pathway of complement activation, *DLS* dynamic light scattering, *DSC*differential scanning calorimetry, *ELISA* enzyme-linked immunosorbent assay, *FACS* fluorescence-activated cell sorting, *FcR* fragment crystallizable receptor, *FcγRIa*Fc gamma receptor Type Ia, *FcγRIIa* Fc gamma receptor Type IIa, *FcγRIIb* Fc gamma receptor Type IIb, *FcγRIIIa* Fc gamma receptor Type IIIa, FcγRIIIb Fc gammareceptor Type IIIb, *FcRn* neonatal Fc receptor, *FFF* field flow fractionation, *FTIR* Fourier-transform infrared spectroscopy, *HC* heavy chain, *HCP* host cell protein,*HIAC* high accuracy light obscuration, *HILIC* hydrophilic interaction liquid chromatography, *UHPLC* untra high performance liquid chromatography *HPLC* highperformance liquid chromatography, *HUVEC* human umbilical vein cells, *LC* light chain, *mbTNF* membrane bound tumor necrosis factor, *MFI* micro-flow imaging,*NUV-CD* near-ultraviolet circular dichroism, *nrCE-SDS* non-reduced capillary electrophoresis–sodium dodecyl sulfate, *PBMC* peripheral blood mononuclear cell,*rCE-SDS* reduced capillary electrophoresis–sodium dodecyl sulfate, *SE-HPLC-SLS* size exclusion high performance liquid chromatography with light scattering, *SE-HPLC* size exclusion high performance liquid chromatography, *SPR* surface plasmon resonance, *sTNF* soluble tumor necrosis factor

### Primary Structure

**Fig. 1:**
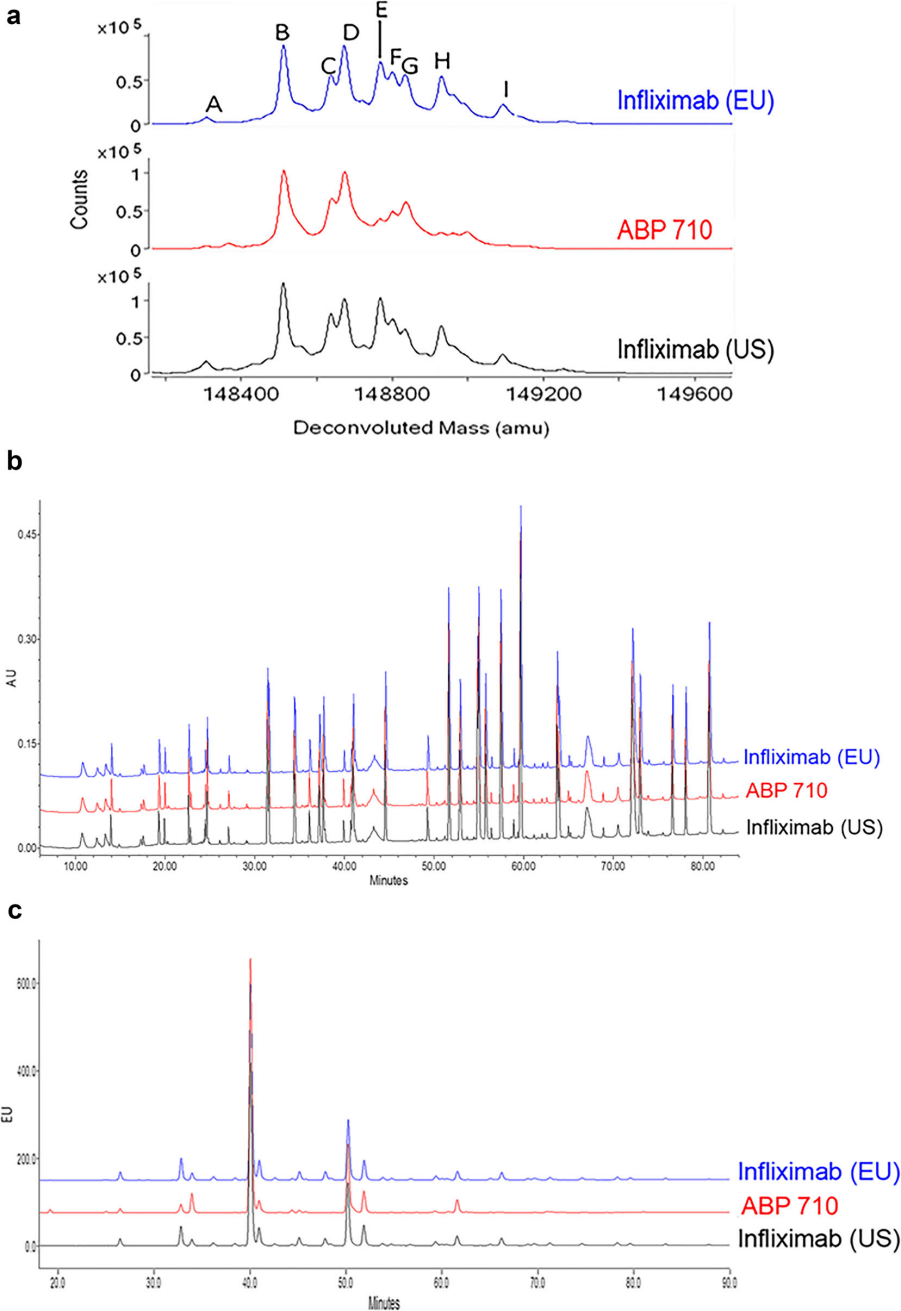
Primary structure assessment of infliximab (EU), ABP 710, and infliximab (US) **(a)** Intact molecular mass. Identified peaks are reported in Table II; **(b)** Reduced tryptic peptide map; **(c)** HILIC glycan map.

ABP 710 and infliximab RP were subjected to intact molecular mass analysis. The deconvoluted intact molecular mass profiles for ABP 710, infliximab (US), and infliximab (EU) are overlaid in Fig. 1a. The differences between the observed masses and the theoretical values are provided in Table II. The theoretical mass calculations were based on the expected amino acid sequence of the RP and masses of the predominant glycan species. The predominant species for ABP 710, infliximab (US), and infliximab (EU) are consistent with the presence of 2 core-fucosylated complex N-glycans with either 0 or 1 terminal galactose residue. Peaks A, B, C and E consist of two core-fucosylated complex N-glycans with no terminal galactose residue. Peaks D, F, G, H and I are molecules with glycans containing 0, 1 or 2 terminal galactose residues. ABP 710 and infliximab (US and EU) contain incompletely processed C-terminal lysine on the heavy chain (HC). The molecular masses for peaks A, B, D and G correspond to molecules with no C-terminal lysine residues on the HC. The molecular masses for structures containing 1 C-terminal lysine residue was confirmed for peaks C and F, and the molecular masses for structures containing 2 C-terminal lysine residues were confirmed for peaks E, H and I. The observed molecular masses for ABP 710 and infliximab (US) are similar, and all the peaks (A, B, C, D, E, F, G, H and I) are within ±30 ppm of their theoretical masses which are well within the method and instrument capability of ±100 ppm accuracy. The results confirm that the products have the same amino acid composition and similar intact molecular masses. However, ABP 710 has lower abundances of peaks E, H and I than infliximab (US) due to slightly lower levels of C-terminal lysine. The C-terminal lysine level difference was confirmed by reduced and deglycosylated molecular mass analysis (see below). ABP 710 and infliximab RP are administered intravenously, and lysine residues, when present, are cleaved by carboxypeptidases within minutes; hence, this minor difference is not considered clinically meaningful (29–31). The results demonstrate that ABP 710 has similar intact molecular masses compared to infliximab RP.

**Table II:** Analytical Similarity Assessment between ABP 710 and Infliximab

**Analytical Testing/Attributes**	**ABP 710 (n)**	**Infliximab US (n)**	**Infliximab EU (n)**
Intact molecular weight (ppm^a^)			
Peak A: A1G0F:A2G0F, 148,310 Da	5 (1)	5 (1)	0 (1)
Peak B: A2G0F:A2G0F, 148,514 Da	2 (1)	11 (1)	6 (1)
Peak C. A2G0F:A2G0F + 1 K, 148642 Da	3 (1)	21 (1)	20 (1)
Peak D: A2G0F:A2G1F, 148,676 Da	5 (1)	12 (1)	13 (1)
Peak E: A2G0F:A2G0F + 2 K, 148770 Da	10 (1)	8 (1)	5 (1)
Peak F: A2G0F:A2G1F + 1 K, 148804 Da	10 (1)	16 (1)	18 (1)
Peak G: A2G1F:A2G1F or A2G0F:A2G2F, 148,838 Da	7 (1)	29 (1)	20 (1)
Peak H: A2G0F:A2G1F + 2 K, 148932 Da	9 (1)	8 (1)	2 (1)
Peak I: A2G1F:A2G1F or A2G0F:A2G2F + 2 K, 149094 Da	0 (1)	10 (1)	3 (1)
Reduced and deglycosylated HC, 49389 Da	4 (1)	17 (1)	22 (1)
Reduced and deglycosylated HC + K, 49517 Da	4 (1)	24 (1)	18 (1)
Reduced and deglycosylated LC, 23439 Da	3 (1)	26 (1)	27 (1)
Glycan Map (%)			
High Mannose	3.2–4.2 (14)	3.0–5.5 (28)	3.1–5.5 (20)
Afucosylation	6.1–7.3 (14)	6.7–11.1 (28)	6.9–10.9 (20)
β-galactosylation	25.1–34.2 (14)	25.9–52.0 (28)	25.7–51.2 (20)
α-galactosylation	N/A	1.6–6.6 (28)	1.5–6.1 (20)
Sialylation	0.9–1.6 (14)	3.1–10.0 (28)	4.1–9.7 (20)
Isoelectric point	7.4 (1)	7.4 (1)	7.4 (1)
FTIR/spectral similarity (%)			
US RP	99.7–99.8 (6)	99.7–100 (3)	99.7–99.9 (3)
EU RP	99.6–99.9 (6)	99.7–99.9 (3)	99.6–100 (3)
NUV-CD/spectral similarity (%)			
US RP	97.5–99.1 (6)	98.9–100 (3)	98.7–99.2 (3)
EU RP	97.1–99.3 (6)	98.2–98.7(3)	98.3–100 (3)
DSC (°C)			
T_m1_	67.7–67.9 (9)	67.6–67.8 (6)	67.6–67.8 (6)
T_m2_	83.3–84.3 (9)	83.3–84.2 (6)	83.2–84.3 (6)
Size Variants by SE-UHPLC (%)			
Main Peak	98.6–99.2 (12)	99.3–99.8 (28)	99.5–99.8 (19)
Size Variants by rCE-SDS			
Heavy Chain + Light Chain	96.6–97.3 (12)	98.2–98.9 (28)	98.4–98.9 (19)
Fragments	0.7–1.0 (12)	0.3–1.1 (28)	0.4–0.8 (19)
Non-glycosylated heavy chain	1.6–2.1 (12)	0.4–0.6 (28)	0.4–0.6 (19)
Size Variants by nrCE-SDS			
Main Peak	97.1–98.1 (12)	97.8–98.9 (28)	98.4–98.9 (12)
Charge Variants by CEX-UHPLC			
Main Peak	43.5–47.7 (12)	31.7–51.7 (28)	37.2–45.4(18)
Acidic Peaks	19.5–23.6 (12)	9.9–15.8 (28)	9.6–17.7 (18)
Basic Peaks	30.0–36.1 (12)	34.2–56.2 (28)	40.5–53.2 (18)
HIAC subvisible particle (particles/mL)			
≥ 2 μm	40–569 (19)	4568–7064 (7)	3782–14,353 (7)
≥ 5 μm	5–138 (19)	724–1341 (7)	675–3138 (7)
≥ 10 μm	0–39 (19)	64–172 (7)	82–315 (7)
≥ 25 μm	0–2 (19)	0–3 (7)	1–3 (7)
MFI subvisible particle (particles/mL)			
≥ 5 μm particles	19–1260 (19)	952–3263 (7)	758–6047 (7)
≥ 5 μm non-spherical particles	12–260 (19)	328–1940 (7)	384–3425 (7)
SV-AUC/monomer (%)	98.4–99.4 (3)	98.8–99.7 (3)	99.1–99.9 (3)
SE-HPLC-LS MW (kDa)			
Monomer	150–151 (3)	151–152 (3)	151–152 (3)
Dimer	284–287 (3)	292–319 (3)	285–334 (3)
Reconstituted protein concentration (mg/mL)	9.1–9.6 (19)	9.2–9.8 (26)	9.1–9.7 (18)
Protein content (mg)	96–99 (19)	97–102 (26)	96–101 (18)

n = number of lots tested

^a^ppm = 1,000,000 x |(observed mass - theoretical mass)|/theoretical mass

Glycosylation status of observed monoclonal antibody species: A = number of antenna (GlcNAc) on trimannosyl core; F = Fucose; G = linked galactose on antenna; *HC* heavy chain, K = C-terminal lysine. *LC* light chain

Reconstituted protein concentration and protein content determined from 100 mg vial

*SV-AUC* sedimentation velocity analytical ultracentrifugation, *CEX-UHPLC* cation exchange ultra-high performance liquid chromatography, *DSC* differential scan-ning calorimetry, *FTIR* Fourier-transform infrared spectroscopy, *HC* heavy chain, *LC* light chain, *MFI* micro-flow imaging, *NUV-CD* near-UV circular dichroism, *nrCE-SDS* non-reduced capillary electrophoresis–sodium dodecyl sulfate, *NGHC* non-glycosylated heavy chain, *rCE-SDS* reduced capillary electrophoresis–sodium dodecyl sulfate, *RP* reference product, *SE-UHPLC* size exclusion untra high performance liquid chromatography, *SE-HPLC-LS* size exclusion high performance liquid chromatography with light scattering

ABP 710 and infliximab RP were also subjected to analysis by mass spectrometry after reduction and deglycosylation. The deconvoluted HC and light chain (LC) mass profiles were similar for both ABP 710 and infliximab RP; and there were no new species observed in the profiles for the reduced deglycosylated ABP 710 when compared to infliximab RP (data not shown). The differences between the observed masses and the theoretical values are provided in Table II. The theoretical mass calculations were based on the expected amino acid sequence. The major peaks correspond to the expected theoretical masses of HC and LC. The observed molecular masses for ABP 710 and infliximab (US) were within 27 ppm of the theoretical masses expected for infliximab RP which are well within the method and instrument precision of ±100 ppm accuracy. The slightly lower abundance of HC with unprocessed C-terminal lysine in ABP 710 is not clinically meaningful. The results demonstrate that the reduced and deglycosylated HC and LC masses of ABP 710 are similar to those of infliximab (US).

The reduced peptide map was used to compare the amino acid sequence and identify post-translational modifications. The tryptic peptide map chromatograms for the side-by-side comparison of ABP 710, infliximab (US) and infliximab (EU) are overlaid in Fig. 1b. The observed molecular masses for the ABP 710 and infliximab (US) tryptic peptides were within ±50 ppm of the theoretical masses expected for infliximab RP (data not shown). The peptide map profiles were visually similar, and no new peaks above the limit of detection calculated based on system noise were detected in ABP 710 compared to infliximab (US). The same low level post-translational modifications were observed in both ABP 710 and infliximab RP, including methionine oxidation and asparagine deamidation commonly detected in recombinant monoclonal antibodies. Approximately 1% to 3% methionine oxidation in the Fc region and approximately 1% to 2% asparagine deamidation in the HC CDR and Fc region were observed for ABP 710 and infliximab RP. These low level chemical modifications are stable in the lyophilized drug products (see *Thermal stability and forced degradation* below), and any minor differences had no impact on biological functions and would not result in a clinically meaningful difference. Both ABP 710 and infliximab RP are IgG1 monoclonal antibodies that contain a total of 16 disulfide bonds, including 12 intrachain and 4 interchain. The same disulfide structure for ABP 710 and infliximab RP was confirmed by non-reduced peptide map as expected for IgG1 disulfide structure. Overall, the results support the conclusion that ABP 710 has the same amino acid sequence with minor differences in the post-translational modifications compared to infliximab RP.

A comparison of the glycan map chromatograms for ABP 710, infliximab (US) and infliximab (EU) lots is provided in Fig. 1c. As stated in the introduction, infliximab RP is manufactured in a SP2/0 (mouse myeloma) cell line, whereas ABP 710 is manufactured using CHO cells. Unlike infliximab (US), ABP 710 does not contain detectable levels of non-human glycans, such as NGNA and α-Gal. ABP 710 contains NANA sialylation, at minor levels, which is absent in infliximab (US). It is known that the use of differing expression systems (CHO *vs* SP2/0) will result in differences in the glycan profile of the expressed protein, which may or may not be clinically meaningful. Additionally, previously published work elucidated the complex structure-function relationship of multiple glycan attributes, i.e., afucosylation, high mannose, and beta-galactosylation for their influence on FcγRIIIa-binding, ADCC and CDC (16). Recognizing this, the manufacturing process for ABP 710 was carefully designed, with emphasis on maintaining similar biological function and minimizing any potential impact on safety, immunogenicity and efficacy. The following glycan groups were evaluated as part of the similarity assessment, based on their potential to impact PK and/or effector functions: afucosylation, high mannose, galactosylation and sialylation.

High mannose levels were calculated as the sum of high mannose glycans, M5 through M7. Results showed that ABP 710 is similar to infliximab RP for high mannose levels (Table II). Afucosylated glycans are comprised of afucosylated complex type and afucosylated hybrid type glycans. ABP 710 lots have consistent, but slightly lower levels of afucosylated glycans compared to the infliximab RP ranges (Table II). Importantly, ABP 710 is similar to infliximab RP for biological functions, including functions influenced by afucosylated glycans, namely binding to FcγRIIIa and ADCC activity (Table III). The level of β-galactosylation was calculated as the sum of all complex type and hybrid type glycan structures which contain at least one terminal β-galactose and no terminal α-galactose. Results showed that ABP 710 is similar to infliximab RP for β-galactosylation (Table II). Infliximab RP has low levels of α-galactosylation (≤ 6.6%), and ABP 710 has no α-galactosylation (Table II). In addition, the level of sialylation in ABP 710 and infliximab RP are provided in Table II. The level of NANA sialylation in ABP 710 is ≤1.6% and the level of NGNA sialylation in infliximab (US) is ≤10.0%.

**Table III:** Summary of Biological Activity Attributes for ABP 710, Infliximab (US), and Infliximab (EU)

**Analytical Testing/Attribute**	**ABP 710 Range (n)**	**Infliximab US Range (n)**	**Infliximab EU Range (n)**
Inhibition of sTNFα-induced apoptosis in U937 (potency) (%)	87–112 (14)	78–117 (28)	89–118 (19)
Binding to sTNFα by ELISA (%)	88–109 (14)	88–112 (25)	98–104 (16)
Binding to sTNFα by SPR (%)	92–109 (10)	90–105 (10)	89–103 (10)
Binding kinetics to sTNFα by SPR (Kd, pM)	113–135 (6)	110–144 (6)	117–142 (6)
Inhibition of sTNFα-induced IL-8 release in HUVEC (%)	97–102 (3)	98–116 (3)	92–101 (3)
Inhibition of sTNFα-induced cell death in L929 (%)	94–104 (3)	100–119 (3)	92–98 (3)
Binding to mbTNFα on CHO MT-3 cells by imaging cytometry (%)	92–108 (14)	91–114 (25)	97–113 (16)
Reverse signaling in Jurkat mbTNFα cell line (%)	95–109 (14)	90–114 (24)	96–107 (16)
Binding to FcγRIIIa (158V) by SPR (%)	98–117 (10)	82–105 (10)	89–110 (10)
Binding kinetics to FcγRIIIa (158V) by SPR (Kd, nM)	68–80 (10)	63–93 (10)	59–89 (10)
Binding to FcγRIIIa (158F) by SPR (%)	88–119 (10)	86–108 (10)	87–106 (10)
NK92 ADCC activity (%)	94–-133 (14)	80–169 (27)	100–166 (20)
PBMC ADCC activity (%)	90–118 (10)	82–119 (10)	86–41 (10)
Binding to C1q by ELISA (%)	83–104 (14)	60–93 (25)	61–95 (17)
CDC activity (%)	93–110 (14)	93–136 (27)	93–135 (19)
Binding to FcγRIIa (131R) by SPR	101–104 (3)	98–99 (3)	100–105 (3)
ADCP activity	97–104 (3)	94–116 (3)	95–106 (3)
Binding to FcγRIa by AlphaLISA	92–99 (3)	92–95 (3)	92–101 (3)
Binding to FcγRIIb by SPR	91–105 (3)	91–99 (3)	96–110 (3)
Binding to FcγRIIIb by SPR	96–117 (10)	91–111 (10)	87–106 (10)
Binding to FcRn by AlphaScreen (%)	90–123 (14)	92–108 (27)	87–112 (20)
Binding to FcRn by SPR (%)	91–109 (10)	93–134 (10)	84–137 (10)

n = number of lots tested; all relative activity (%) was calculated against ABP 710 reference standard lot

*ADCC* antibody-dependent cell-mediated cytotoxicity; *ADCP* antibody-dependent cellular phagocytosis; *CDC* complement-dependent cytotoxicity; *CHO* Chinese Hamster Ovary cell; *FcRn* Fc neonatal receptor; *ELISA* enzyme-linked immunosorbent assay; *HUVEC* human umbilical vein cells; *infliximab (EU)* European Union-authorized infliximab; *infliximab (US*) United States Food and Drug Administration-licensed infliximab; *mbTNF* membrane bound tumor necrosis factor; *n* number of batches; *PBMC* peripheral blood mononuclear cell; *SPR* surface plasmon resonance; *sTNF* soluble tumor necrosis factor; *TNF* tumor necrosis factor; *TNFα* tumor necrosis factor-α

### Higher Order Structure

**Fig. 2:**
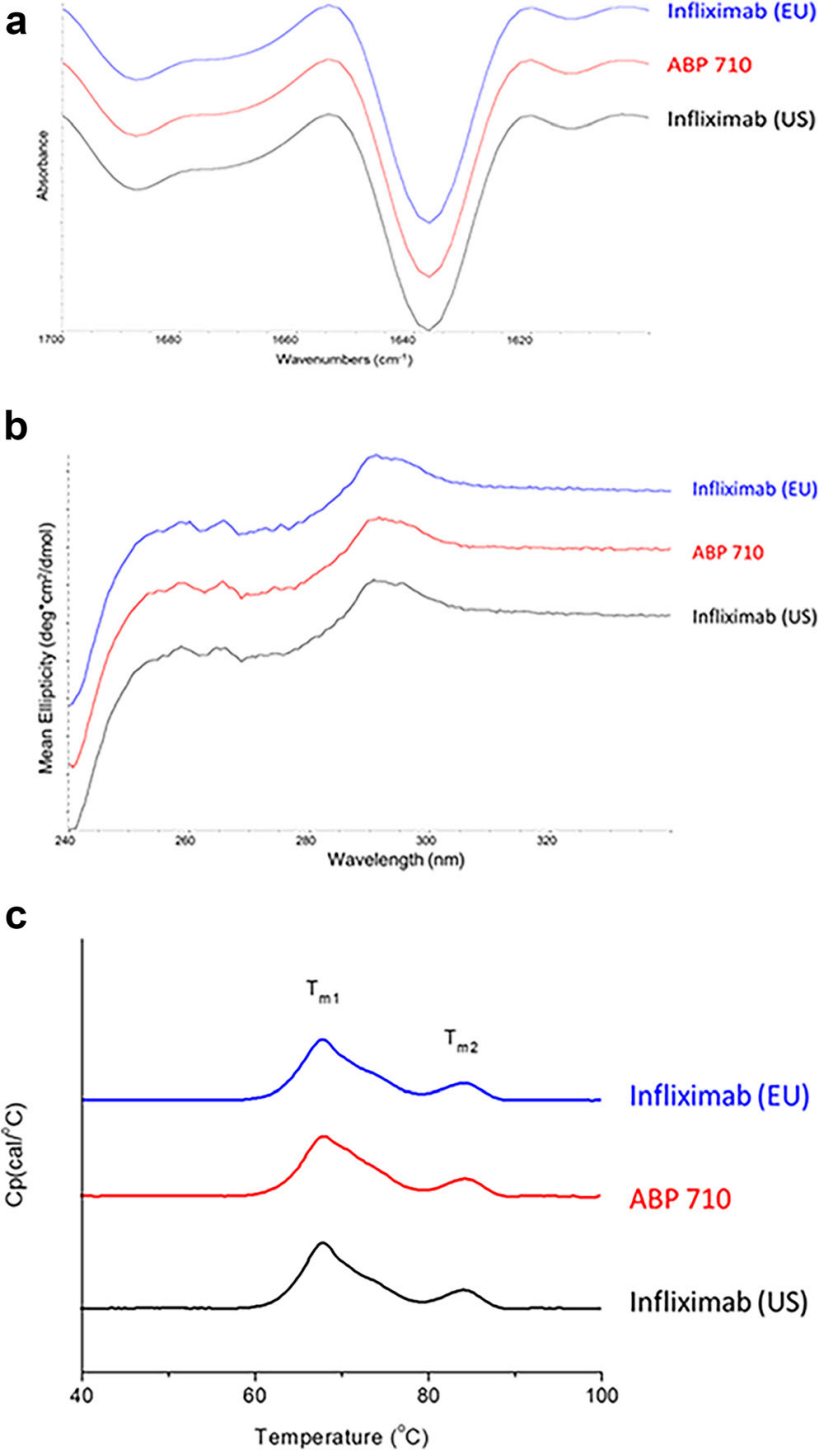
Higher order structural assessment of infliximab (EU), ABP 710, and infliximab (US). **(a)** Second derivative FTIR spectra; **(b)** NUV CD spectra; **(c)** DSC thermograms.

ABP 710 and infliximab RP were subjected to FTIR spectroscopy analysis to assess the secondary structure similarity. The second derivative FTIR spectra for ABP 710 and infliximab (US) are shown in Fig. 2a. The ABP 710 and infliximab RP profiles are visually similar and exhibit a strong β-sheet band at around 1639 cm^−1^ together with a β-sheet band at 1689 cm^−1^, indicating the presence of predominantly anti-parallel β-sheet structure typical of antibodies. Spectral similarity of ABP 710, infliximab (US) and infliximab (EU) measured relative to infliximab (US) reference spectrum and infliximab (EU) reference spectrum was quantified. ABP 710 demonstrated >95% spectral similarity relative to infliximab RP (Table II). Altogether, the secondary structures of ABP 710 and infliximab RP are similar by FTIR spectroscopy.

Near UV-CD (NUV-CD) was performed on ABP 710 and infliximab RP to assess tertiary structure similarity. Spectra comparing ABP 710 and infliximab RP are shown in Fig. 2b. The profiles for ABP 710 and infliximab RP are visually similar, and all the spectra contain signals with peaks from tryptophan (255 to 275 nm), tyrosine (275 to 285 nm) and phenylalanine (285 to 295 nm), superimposed on the broad disulfide signal from 250 to 285 nm. The intensity of these features reflects the native structure and demonstrates that the disulfide bonds and aromatic amino acids are in the expected environment consistent with the proper folding of the proteins. Spectral similarity of ABP 710, infliximab (US) and infliximab (EU) measured relative to infliximab (US) reference spectra and infliximab (EU) reference spectra was quantified. ABP 710 demonstrated >95% spectral similarity relative to infliximab RP (Table II). Altogether, the tertiary structures of ABP 710 and infliximab RP are similar by NUV-CD spectroscopy.

ABP 710 and infliximab RP were analyzed by DSC to assess conformation and thermal stability. ABP 710 and infliximab RP have 2 similar endothermic thermal transitions, which correspond to the unfolding of both the antigen binding fragment/HC constant region (C_H_2) (T_m1_) and C_H_3 (T_m2_) domains (Fig. 2c). Thermal transition temperatures of ABP 710 were compared to the thermal transition temperatures of infliximab RP (Table II). The results show that both transition temperatures T_m1_ of ABP 710 and infliximab RP and T_m2_ of ABP 710 and infliximab RP are similar. The DSC results demonstrate that ABP 710 has similar conformation and thermal stability compared to infliximab RP.

### Particles and Aggregates

Particles and aggregates of ABP 710, infliximab (US), and infliximab (EU) were assessed using a combination of test methods. Subvisible particles were assessed quantitatively and qualitatively by HIAC and MFI. Aggregates were assessed by SV-AUC and size-exclusion HPLC coupled with static light scattering (SE-HPLC-SLS) detection.

The levels of subvisible particles were assessed using HIAC™ and MFI™ methods. The HIAC™ method was used to quantify ≥2 μm, ≥ 5 μm, ≥ 10 μm and ≥ 25μm subvisible particles. The subvisible particle results assessed by HIAC™ demonstrate that ABP 710 has lower levels of particles (Table II). Having fewer particles than the RP is considered acceptable as fewer particles are unlikely to negatively impact the safety profile of ABP 710. Overall, by HIAC™, ABP 710 has similar levels of subvisible particles compared to infliximab RP.

The MFI method was used to quantify total particles and non-spherical (proteinaceous) particles. The ≥5 μm subvisible particle results and ≥ 5 μm non-spherical particle results for ABP 710 and infliximab RP are shown in Table II. The levels of ≥5 μm non-spherical particles for ABP 710 are consistent with infliximab RP.

Aggregates of ABP 710 and infliximab RP were assessed by SE-HPLC-SLS and the results are presented in Table II expressed as the molar mass of monomer and HMW species. The main peak observed for ABP 710 shows a molar mass of approximately 150–151 kDa, which is consistent with the molar mass of infliximab (US and EU) monomer (151–152 kDa). The HMW species observed for ABP 710 shows a molar mass of approximately 284–287 kDa, and the HMW species observed for infliximab RP was 292–319 kDa for US and 285–334 kDa for EU RPs. These observed mass ranges are consistent with the theoretical molar mass of the infliximab RP dimer (298 kDa). Hence, the molar masses for the monomer and HMW species of ABP 710 are similar compared to the infliximab RP.

Aggregates were further assessed by SV-AUC. The SV-AUC profiles for ABP 710 are similar to the infliximab RP (data not shown), and the levels of monomer are provided in Table II. The observed HMW species for both ABP 710 and infliximab RP were below the limit of quantification (3.7%) for the assay. Thus, the SV-AUC profile and the levels of HMW species are similar between ABP 710 and the infliximab RP.

### Product-Related Substances and Impurities

Product-related substances and impurities of ABP 710 and infliximab RP were assessed using a combination of methods that evaluate size and charge variants. Physicochemical properties of size variants were assessed by SE-UHPLC, reduced capillary electrophoresis (rCE)-sodium dodecyl sulfate (SDS) and non-reduced CE-SDS nr-CE-SDS. Charge variants were assessed by CEX-HPLC.

A comparison of the SE-UHPLC results for ABP 710, infliximab (US), and infliximab (EU) lots is provided in Table II and Online Resource 1. The ABP 710 lots showed slightly lower levels of main peak compared to infliximab RP. The impurities are primarily made up of HMW species, and no greater than 0.2% of low molecular weight species were detected in all 3 products (data not shown). Characterization of the HMW peak of ABP 710 and infliximab RP by SE-UHPLC-SLS, SV-AUC and denaturing SE-HPLC (data not shown) demonstrated that the HMW peaks for both products is composed of predominantly dimers. In addition, characterization of ABP 710 by denaturing SE-HPLC demonstrated that the HMW is non-covalent and reversible in nature (data not shown). Although the purified HMW species showed decreases in potency and ADCC compared to the main peak (data not shown), the level of HMW in the final lyophilized product is low (≤1.5%) and stable over time. Previously published *in vitro* and *in vivo* studies have shown that dimers at such low levels would have a low risk of impacting immunogenicity (32,33). Finally, results from a clinical study confirmed that ABP 710 has similar safety and immunogenicity compared to infliximab RP (34). Therefore, while the level of HMW species in ABP 710 is higher than in infliximab RP, the difference is not considered to be clinically meaningful and does not impact safety or efficacy.

ABP 710 and infliximab RP were analyzed by reduced CE-SDS (rCE-SDS), in which samples are separated on a capillary into fragments, LC, non-glycosylated heavy chain (NGHC), HC and HMW species. The method is suitable for quantifying fragments, purity (HC + LC), NGHC, and non-reducible HMW species. A comparison of rCE-SDS results for ABP 710, infliximab (US) and infliximab (EU) lots is provided in Table II and Online Resource 2. The ABP 710 lots show slightly lower levels of HC + LC compared to infliximab RP. The difference in HC + LC is less than 2%. This minor difference in HC + LC is primarily due to a higher level of NGHC in ABP 710. The NGHC level is primarily driven by the cell line and the cell culture process. Regardless of this minor difference observed between ABP 710 and infliximab RP, similarity of *in vitro* biological activity, clinical PK and safety of ABP 710 as compared with infliximab RP was maintained. In addition, ABP 710 demonstrated PK equivalence to infliximab RP (data not shown). Lastly, the non-reducible HMW species for ABP 710 and infliximab RP are similar and are at or below the limit of quantitation of the method (0.4%). Overall, these minor differences in purity by rCE-SDS have no impact on biological activity and are not considered clinically meaningful.

ABP 710 and infliximab RP were analyzed by nrCE-SDS, in which samples are separated by capillary electrophoresis into pre-peaks, main peak and post-peaks. The method is suitable for quantifying levels of partially reduced species and fragments (pre-peaks) and main peak. A comparison of nrCE-SDS results for ABP 710, infliximab (US) and infliximab (EU) lots is provided in Table II and Online Resource 3. The results demonstrate that ABP 710 has a slightly lower level of main peak compared to infliximab RP. The impurities are primarily made up of pre-peak species, which represent properly assembled antibodies with one or more broken interchain disulfide bonds (35). These species are known to convert in redox environments, such as those present in physiological conditions, back to fully disulfide-linked antibodies (36). In addition, this minor difference has no impact on the biological activity. Lastly, the level of post-peaks for ABP 710 and infliximab (US) are similar and are below the limit of quantitation of the method (0.4%). Overall, the small quantitative differences in the levels of nrCE-SDS main, pre-peaks and post-peaks for ABP 710 and infliximab RP are not considered clinically meaningful.

**Fig. 3:**
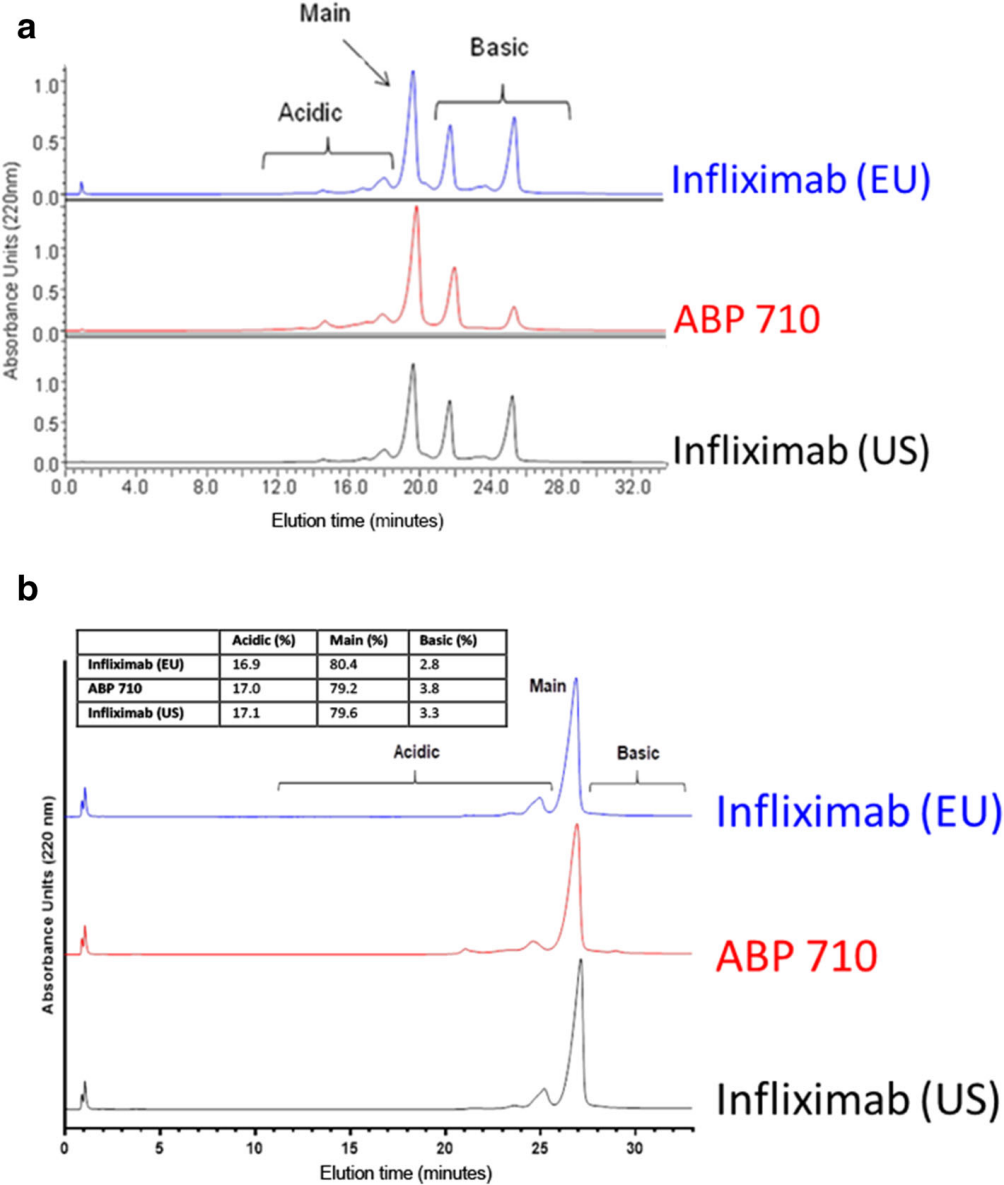
Charge variant assessment of infliximab (EU), ABP 710, and infliximab (US) by CEX-HPLC. **(a)** Untreated; **(b)** carboxypeptidase B digested.

ABP 710 and infliximab RP were separated on a CEX-HPLC column into main peak, acidic peaks and basic peaks. A comparison of the CEX-HPLC results for ABP 710, infliximab (US) and infliximab (EU) lots is provided in Fig. 3a and Table II. Different levels of basic peaks are visually apparent in the CEX-HPLC profiles, which are primarily due to differences in the levels of C-terminal lysine. This was demonstrated by subjecting ABP 710 and infliximab RP to carboxypeptidase B digestion prior to CEX-HPLC analysis (Fig. 3b). Results show that the acidic peaks, main peak, and basic peaks levels are similar between the products upon removal of the C-terminal lysine enzymatically. Additional characterization of the charge variants indicate the acidic peaks contain primarily sialylated glycan species and low level asparagine deamidation species, while the basic peaks contain primarily C-terminal lysine variants and HMW species. The low level chemical modifications such as deamidation and oxidations were observed at the same amino acid residues and at similar levels for both ABP 710 and infliximab RP. However, due to the different glycosylation patterns between Sp2/0 and CHO cell lines, a slightly different distribution of the neutral glycans (afucosylation, high mannose, and galactosylation) in the acidic peaks, main peak, and basic peaks was observed comparing ABP 710 and infliximab RP. Nevertheless, both ABP 710 and infliximab RP show good stability profiles (see *Thermal stability and forced degradation* below) for charge variants, suggesting these chemical modifications and glycan structures are stable in the lyophilized products over time.

### General Properties

ABP 710 and infliximab RP were compared for product strength. Drug product vials were reconstituted, the resulting reconstituted solution volumes were measured gravimetrically by weight and density, and the protein concentrations were determined by UV absorbance at 280 nm. The extinction coefficient was determined experimentally using combinatorial analytical techniques involving amino acid analysis, UV spectrometry, and intact molecular weight analysis, with a resulting experimentally-determined value of within 5% of both the theoretically-determined value and the value reported in the available monograph from the European Pharmacopoeia Commission (37). The reconstituted protein concentration and protein content for all products are shown in Table II. The results confirm that ABP 710 and infliximab RP are highly similar for product strength as demonstrated by reconstituted protein concentration and protein content.

### Biological Activity

**Fig. 4:**
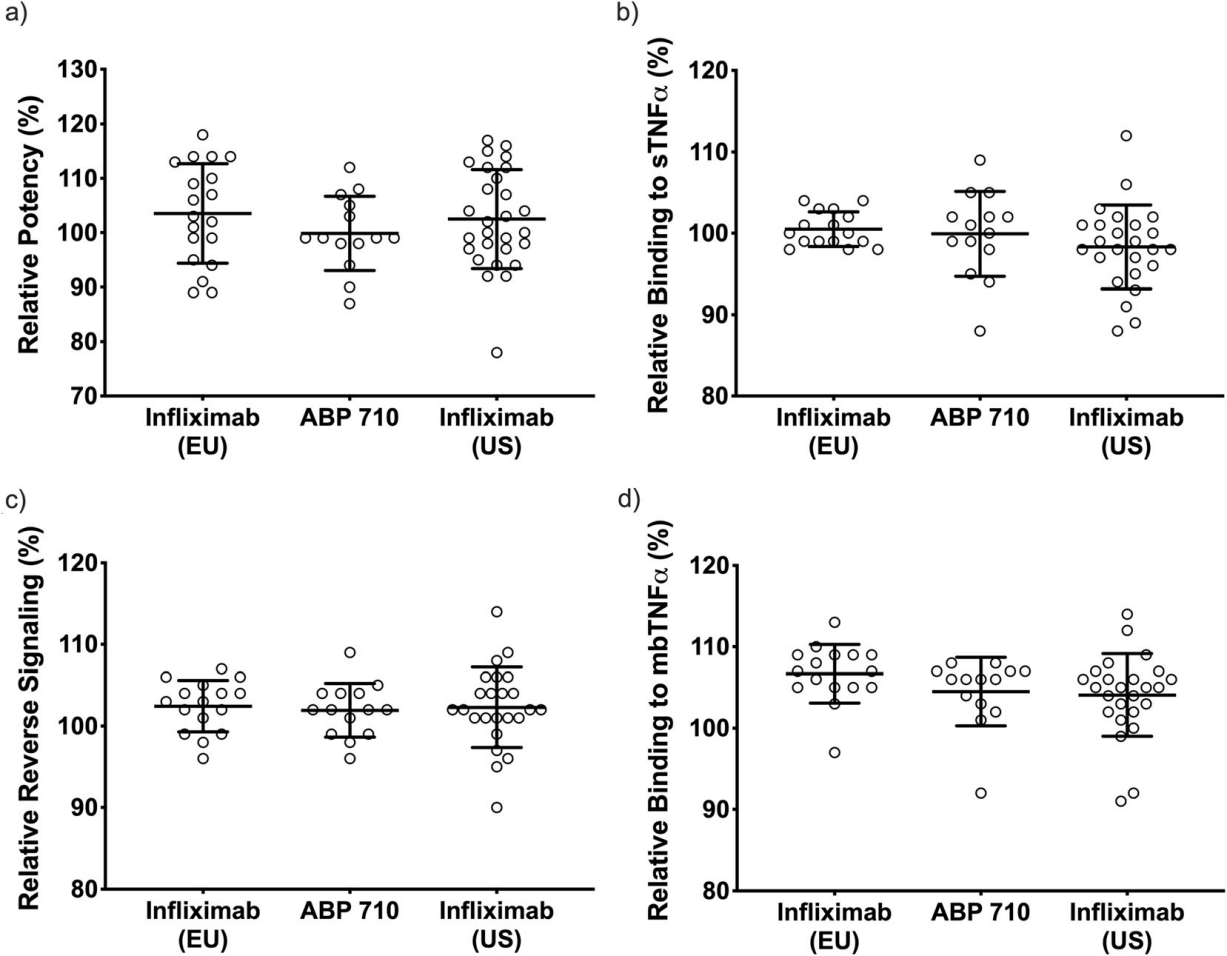
Functional assessment of Fab-mediated activities for ABP 710, infliximab (US) and infliximab (EU). **(a)** Inhibition of sTNFα-induced apoptosis in U937 (potency); **(b)** binding to sTNFα by ELISA; **(c)** reverse signaling; **(d)** binding to mbTNFα on CHO MT-3 cells by imaging cytometry; Individual data points, the mean, and ± one standard deviation are shown.

The primary mechanism of action of infliximab RP and ABP 710 is mediated through binding and neutralization of sTNFα resulting in the suppression of inflammation. The assays selected for the functional similarity assessment were selected to interrogate a wide range of biological functions from multiple angles to assure that any minor biochemical variations would have no significant impact on biological performance of ABP 710 as compared to the RP. The potency assay evaluates the ability of infliximab RP and ABP 710 to inhibit sTNFα-induced apoptosis using the human histiocytic lymphoma cell line U937. The potency assay results are shown in Fig. 4a and Table III, demonstrating that ABP 710 and infliximab RP are similar for the inhibition of sTNFα-induced apoptosis. Related to this mechanism of action, the ability of infliximab RP and ABP 710 to bind sTNFα was also monitored by ELISA and SPR methods. The sTNFα-binding results are shown in Fig. 4b and Table III, demonstrating that ABP 710 is similar to infliximab RP. In addition, sTNF binding kinetics were also conducted to confirm the results observed in the relative binding assays. The source of observed lot-to-lot variability is comprised of variability from manufacturing processes and variability from the analytical testing method. The same analytical method was used to generate results for similarity assessment for both ABP 710 and infliximab RP. However, the manufacturing process variability is uniquely specific to each individual manufacturer’s process and conditions. All analytical methods are qualified per ICH guidelines for accuracy and precision. The target binding and biological activity assays all have an intermediate precision below 10%. All samples were tested in triplicate for each assay, with the mean values reported and CVs averaging around 10%.

**Fig. 5:**
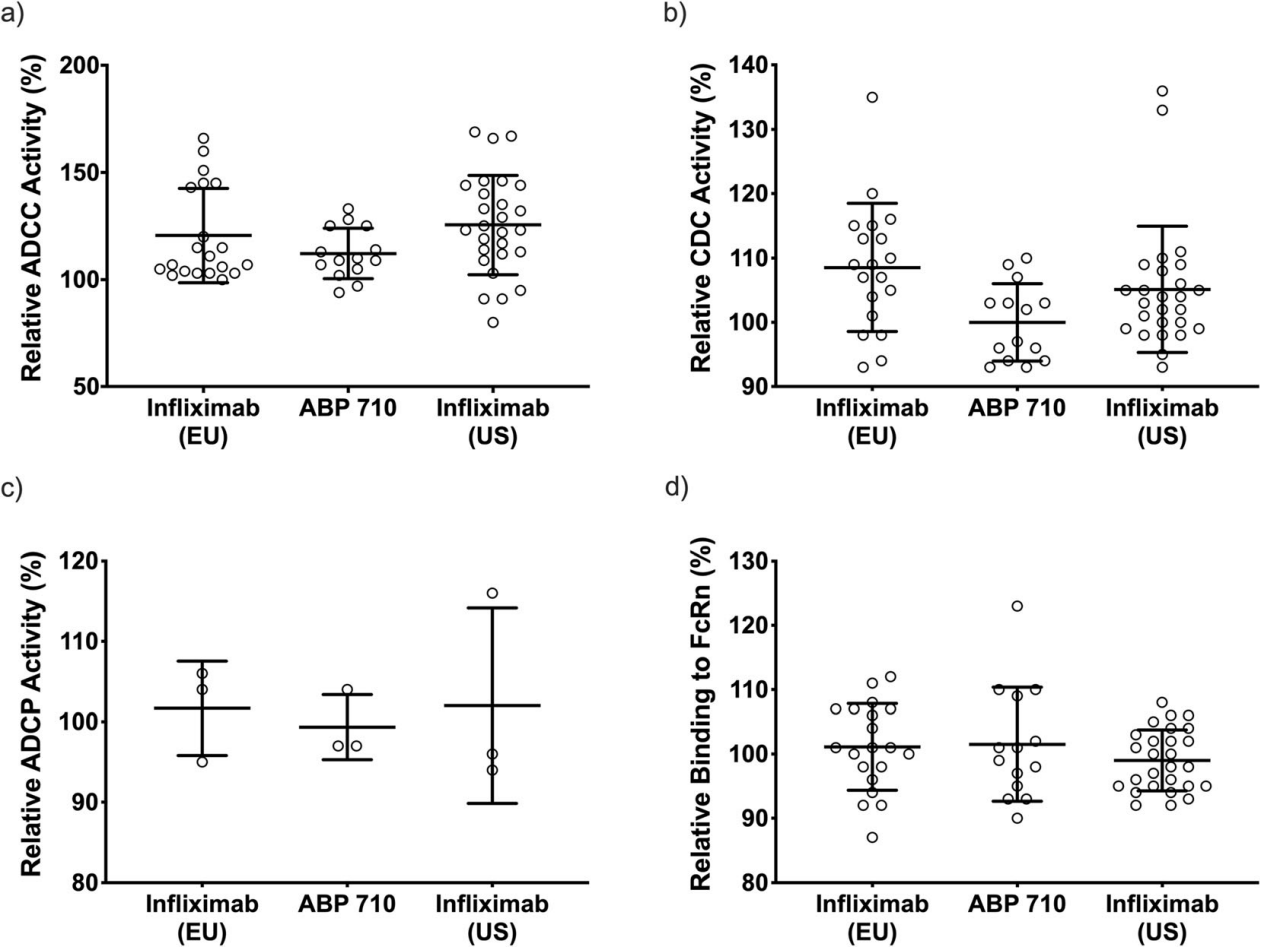
Functional assessment of Fc-mediated activities for ABP 710, infliximab (US) and infliximab (EU). **(a)** NK92 ADCC activity; **(b)** CDC activity; **(c)** ADCP activity; **(d)** binding to FcRn by AlphaScreen; Individual data points, the mean, and ± one standard deviation are shown.

TNFα exists as a membrane bound form (mbTNFα) prior to cleavage by proteases to generate soluble TNFα (sTNFα) (38). Binding to mbTNFα by anti-TNFα antibodies has been shown to induce reverse signaling in some mbTNFα-expressing cell types, leading to apoptosis (2). The induction of apoptosis, in the presence of ABP 710 or infliximab RP, may be involved in clinical efficacy in inflammatory bowel disease (IBD) indications. Accordingly, a reverse signaling assay was developed and performed on ABP 710 and infliximab RP. Results are shown in Fig. 4c and Table III which demonstrate that ABP 710 and infliximab RP are similar for reverse signaling activity. In addition, binding to mbTNFα-expressing cells by infliximab RP and ABP 710 can mediate effector functions (eg, ADCC, ADCP, and CDC) *in vitro*. Binding to mbTNFα by ABP 710 and infliximab (US) and infliximab (EU) was assessed using an assay that measures binding to mbTNFα on CHO MT-3 cells by imaging cytometry. The results are shown in Fig. 4d and Table III, demonstrating that ABP 710 and infliximab RP are similar for mbTNFα binding activity. ABP 710 and infliximab RP were also tested for a number of Fc-mediated activities. ADCC occurs when an antibody binds to an antigen expressed on the surface of cells and the antibody Fc domain simultaneously engages Fc gamma receptors (eg, FcγRIIIa) on the surface of effector cells (eg, natural killer [NK] cells). This leads to the activation of the effector cells with concomitant granule exocytosis and target cell death mediated by perforins and granzyme. ADCC may be important in mediating efficacy of infliximab RP in IBD indications (39). The NK92 ADCC assay was performed on ABP 710 and infliximab RP. The results are shown in Fig. 5a and Table III, demonstrating that ABP 710 is similar to infliximab RP for NK92 ADCC activity.

CDC occurs when an antibody binds to an antigen on the surface of a target cell and the Fc domain of the antibody activates the complement cascade. The hexameric C1q protein binds to the HC constant region (C_H_2) within the Fc domain of IgG molecules, which leads to the formation of the complement complex and results in cell lysis (40). This leads to the formation of the membrane attack complex, which causes pore formation in the target cell membrane and results in cellular lysis. CDC is a mechanism of action that may be important for efficacy in IBD indications (39). The CDC assay was performed on ABP 710 and infliximab RP. The results are shown in Fig. 5b and Table III, demonstrating that ABP 710 and infliximab RP are similar for CDC activity.

ADCP occurs when antibodies bind to antigens expressed on target cells and the antibody Fc domains simultaneously engage Fc receptors on the surface of phagocytic cells. ABP 710 and infliximab RP exhibit ADCP activity with mbTNFα-expressing target cells *in vitro*; however, the relative contribution of ADCP to the clinical efficacy or safety of infliximab RP has not been established. The ADCP assay was performed on ABP 710 and infliximab RP. The results are shown in Fig. 5c and Table III, demonstrating that ABP 710 is similar to infliximab RP for ADCP activity.

FcRn binds to IgG1, IgG2 and IgG4 HCs in the Fc region of IgG molecules. FcRn mediates IgG homeostasis in human adults by maintaining serum IgG levels. An FcRn binding assay by AlphaScreen was performed on ABP 710 and infliximab RP. The results are shown in Fig. 5d and Table III, demonstrating that ABP 710 and infliximab RP are similar for FcRn binding activity.

**Fig. 6:**
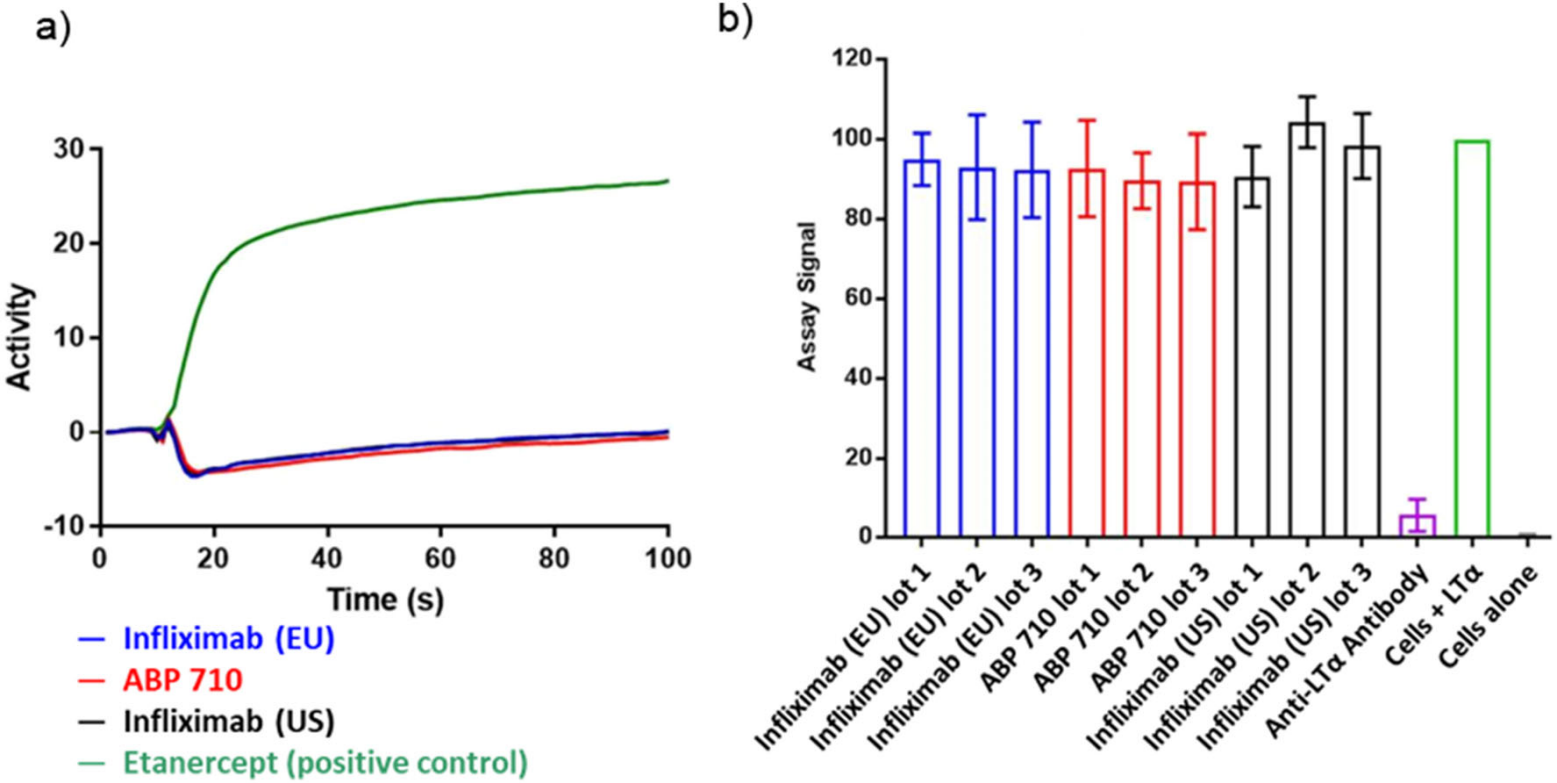
Similarity assessment of the specificity of ABP 710 and infliximab RP. **(a)** Binding of LTα using SPR; **(b)** inhibition of LTα-induced IL-8 release in HUVEC by ABP 710 and infliximab RP.

LTα, a cytokine closely related to TNFα, has been shown to induce signaling through TNFα receptors as well as its own receptors. ABP 710 and infliximab RP are specific for TNFα and do not bind or neutralize LTα. Binding to LTα was tested using SPR to confirm that ABP 710 has a similar binding specificity. Results are presented as side-by-side sensorgrams. ABP 710 and infliximab RP are similar in their lack of binding to LTα as demonstrated in the LTα SPR binding assay (Fig. 6a). Additionally, an LTα-induced IL-8 release in a HUVEC bioassay was performed to further confirm lack of binding to LTα. ABP 710 and infliximab RP demonstrated a lack of inhibition of LTα-induced IL-8 release in HUVEC (Fig. 6b). In addition, the positive control, an anti-LTα antibody, showed the expected inhibition of LTα-induced IL-8 release in HUVEC (Fig. 6b). Therefore, ABP 710 and infliximab RP are considered similar for lack of inhibition of LTα-induced IL-8 release in HUVEC. ABP 710 and infliximab RP were assessed for similarity by additional Fc functional activity assays, including FcγRIa, FcγRIIa(131R), FcγRIIb, FcγRIIIa(158V), FcγRIIIa(158F), FcγRIIIb, and C1q binding. The ranges for the results of ABP 710 and infliximab RP for all biological functional assays are displayed in Table III. For all functional attributes assessed, ABP 710 and infliximab RP show similar activities related to the mechanisms of action relevant for clinical efficacy for all indications.

### Thermal Stability and Forced Degradation

**Fig. 7:**
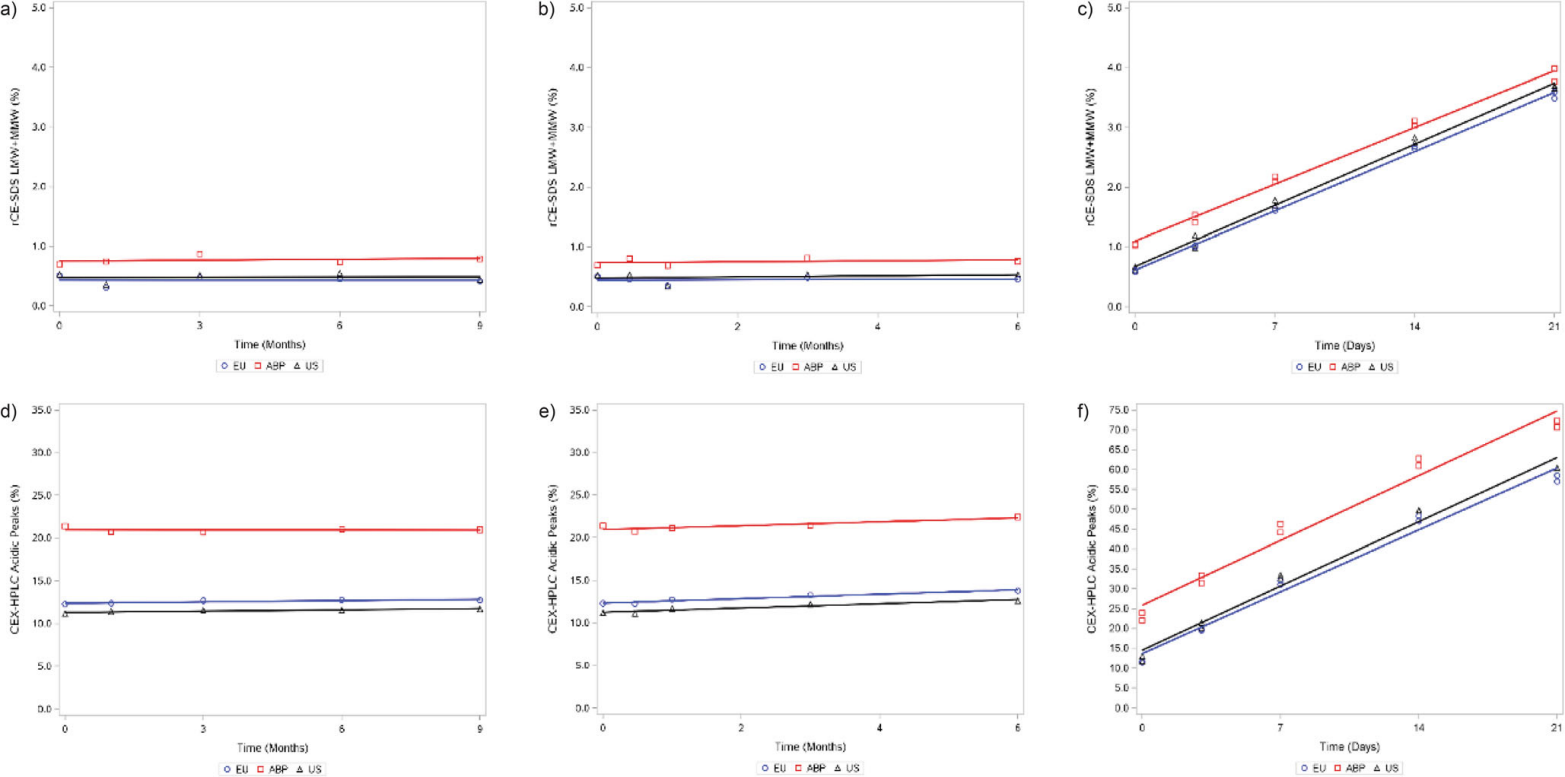
Thermal stability and forced degradation of ABP 710 and infliximab RP. Fragmentation (LMW + MMW) results by rCE-SDS are shown for **(a)** accelerated thermal stability at 25°C; **(b)** stressed thermal stability at 40°C; and **(c)** reconstituted drug product at 40°C. Acidic charge variants results by CEX-HPLC are shown for **(d)** accelerated thermal stability at 25°C; **(e)** stressed thermal stability at 40°C; and **(f)** reconstituted drug product at 40°C.

As part of the analytical similarity assessment, the degradation pathways were evaluated by a set of thermal degradation studies, including accelerated (25°C) and stressed (40°C) stability studies on the lyophilized drug product of ABP 710, infliximab (US) and infliximab (EU). Additionally, a thermal degradation study was performed at 40°C on reconstituted drug product to aid in the comparison of ABP 710 and infliximab RP degradation profiles in solution. Thermal stress condition for reconstituted products at 40°C was selected because development studies indicated 50°C incubation of the lyophilized product resulted in insufficient degradation and temperatures slightly above 60°C resulted in a discolored and collapsed lyophilized cake. To compare degradation rate between ABP 710 and infliximab RP, protein fragmentations measured by rCE-SDS and chemical modification, including deamidation and oxidation, measured by CEX-HPLC are shown in Fig. 7. The lyophilized ABP 710 and infliximab RP products have good stability at 25°C for up to 9 months and at 40°C for up to 6 months with minimal changes in fragmentation and chemical modification, with no meaningful degradation. On the other hand, meaningful degradation rates were observed for the reconstituted solution at 40°C for up to 21 days; these rates were similar for ABP 710 and infliximab RP. Overall, ABP 710 has similar thermal stability and degradation rates compared to infliximab (US) and infliximab (EU).

## Discussion

This study was designed to evaluate the similarity of the proposed biosimilar ABP 710 to infliximab RP. Comparisons were made using RP acquired from 2 sources, infliximab (US) and infliximab (EU), by assessing through a comprehensive structural and functional comparison. Similarity for the primary structures between ABP 710 and infliximab (US) and infliximab (EU) were investigated using several complementary characterization methods, including intact protein mass analysis, reduced and deglycosylated HC and LC mass analysis, reduced and non-reduced peptide mapping and glycan mapping, as well as purity assays to assess size and charge characteristics of the molecules. Some minor differences in physiochemical attributes were observed between ABP 710 and infliximab RP, primarily due to the cell line difference between ABP 710, expressed from CHO, and infliximab RP, expressed from Sp2/0. Apparent differences in some PTMS, such as glycan profile and charge variants, were also likely due to the unique host cell line used in the production of ABP 710. Therefore, a comprehensive biological characterization using multiple orthogonal assays to probe the different intracellular forward-signaling pathways, such as induction of apoptosis and cytokine release, was critical to demonstrate similarity for biological activities between ABP 710 and infliximab RP. Infliximab RP is approved for multiple indications, and the biological functions required for clinical efficacy are likely complex and may not be the same across all indications (41–43).The biological and functional similarity results show that minor physicochemical differences do not affect biological functions relevant to the mechanism of action of ABP 710 and infliximab RP. The results also demonstrate that infliximab sourced from both the US and EU are analytically comparable.

Results from reduced peptide mapping demonstrate that the amino acid sequence of ABP 710 is identical to that of infliximab RP, whereas similarity in post-translational modifications was demonstrated through MS and glycan mapping. Asparagine deamidation and methionine oxidation were observed at similar levels (≤ 3%) for ABP 710 and infliximab RP. As a result of the different host cell lines, some post-translational modification differences are noted. For example, ABP 710 does not contain α-galactosylation and NGNA sialylation, which are non-human glycans commonly seen in Sp2/0 cell line-expressed glycoproteins. The levels of these non-human glycans in infliximab RP are low, and there is no reported evidence of any adverse event directly associated with the presence of these non-human glycans in infliximab RP. Therefore, the absence of these non-human glycans in ABP 710 is not expected to impact clinical efficacy and safety. In fact, the comparative clinical studies demonstrated similarity of ABP 710 with infliximab RP for PK and safety (34,44). The safety risks or even benefits may vary and could be influenced by an individual’s prior exposure and pre-existing antibodies against these non-human glycans. Considering the chronic nature of these diseases and treatments, the absence of these non-human glycans in ABP 710 may potentially result in an improved long-term immunogenicity profile for patients. Due to the limitations of the comparative clinical study design as required specifically for biosimilar regulatory approvals, the potential impact on long-term safety and immunogenicity due to these non-human glycans on a large and diverse population remains to be determined.

The analysis of the higher order structure confirmed that ABP 710 and infliximab RP have similar secondary and tertiary structure and overall conformational stability. Additionally, thermal forced degradation of both lyophilized powder and reconstituted solution was studied. Overall, ABP 710 finished product and infliximab RP were demonstrated to have similar stability profiles.

The similarity of ABP 710 and infliximab RP with regard to *in vitro* binding to FcRn and FcγRIIIa and *in vitro* effector function activities, ADCC and CDC, was also demonstrated. TNF-binding similarity was demonstrated through multiple sensitive biological characterization assays, including binding to sTNFα by enzyme-linked immunosorbent assay (ELISA), binding to mbTNFα by a competitive imaging cytometry-based assay, inhibition of sTNFα-induced apoptosis in the U937 cell line and reverse signaling via induction of apoptosis in mbTNFα-expressing Jurkat cells. Furthermore, an extensive panel of Fc receptor-binding and Fab and Fc-mediated effector actvities was also performed to support the overall conclusion of similarity of ABP 710 and infliximab RP.

## Conclusion

ABP 710 has been developed as a biosimilar to infliximab RP. Results of this comprehensive analytical evaluation of quality attributes and biological functions are established for the primary structure, higher order structure, particles and aggregates, product-related substances and impurities, biological activities and general properties. Despite the minor analytical differences observed for several biochemical attributes, including post-translational modifications expected from the cell line difference, a comprehensive panel of biological assays was selected to interrogate a wide range of biological functions from multiple angles to assure that any minor biochemical variations would have no significant impact on biological performance. All biological functions, including target binding, Fc-mediated binding, and Fab + Fc mediated effector functions, were similar compared to infliximab RP. Further, similarity studies in clinical PK and clinical safety and efficacy have been conducted and corroborate similarity of ABP 710 with infliximab RP (34,41). The totality of evidence supports that ABP 710 is highly similar to infliximab RP, and these results supported the approval of ABP 710 in the United States (Avsola™, Amgen Inc., Thousand Oaks, CA). In conclusion, the analytical similarity assessment results provide the foundation for the scientific justification of examination of clinical similarity within an indication as well as for the extrapolation of the indications approved for infliximab RP to ABP 710. Furthermore, this similarity assessment provides confidence for similarity of clinical safety and efficacy in patients.

### Acknowledgments and Disclosures

This work was funded by Amgen Inc. Medical writing support was provided by Sonya G. Lehto, PhD (Amgen Inc.) and Monica Ramchandani, PhD (Amgen Inc.). Thanks to Dipanwita Batabyal, Teresa Born, Shawn Cao, Jane Dankberg, Chih-Kai Fang, Nancy Jiao, Palanisamy Kanakaraj, Sheeba Kazi, Dayong Qiu, Chris Rollins, Bob Sandrock, Renuka Sivendran, Nithya Srinivasan, and Jie Wen for excellent scientific and experimental support. Authors are or were employees and stockholders of Amgen Inc. during the time of this research. This article does not contain any studies with human participants or animals. All laboratory health and safety procedures have been complied with in the course of conducting this experimental work.

## Electronic supplementary material

ESM 1 (PNG 388 kb)

High resolution image (TIF 487 kb)

ESM 2 (PNG 565 kb)

High resolution image (TIF 580 kb)

ESM 3 (PNG 563 kb)

High resolution image (TIF 615 kb)

ESM 4 (DOCX 15 kb)
