# Supplementary figures and images for: Analytical and Functional Similarity Assessment of ABP 710, a Biosimilar to Infliximab Reference Product
Source: Pharm Res. 2020 May 31;37(6):114. doi: 10.1007/s11095-020-02816-w (PMC7261735; doi:10.1007/s11095-020-02816-w)

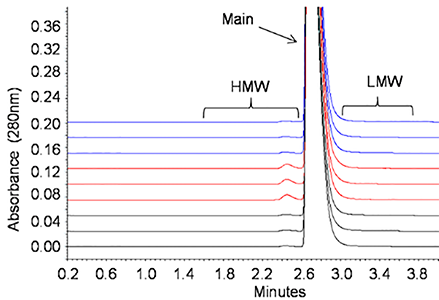

Supplement: Supplementary file 1 — (PNG 388 kb) [file 11095_2020_2816_Fig8_ESM.png]

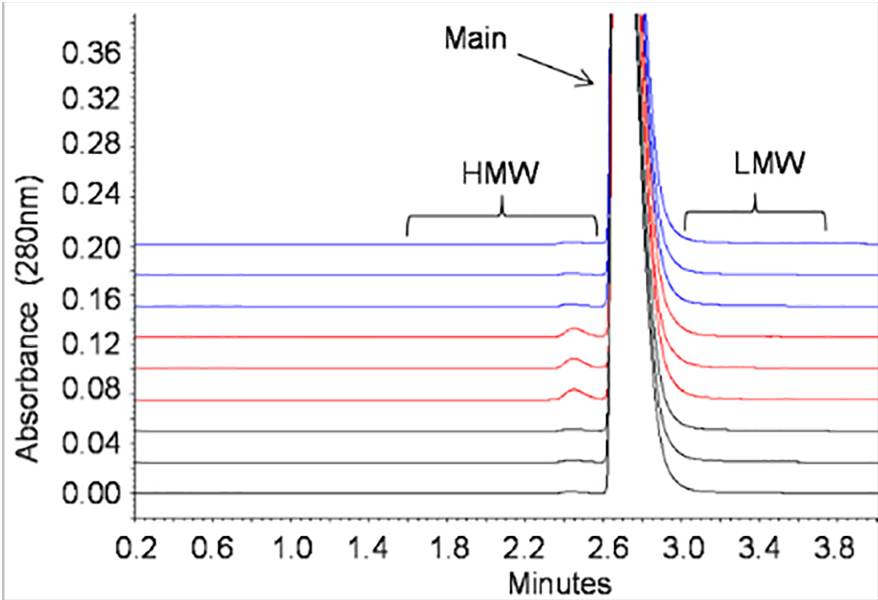

Supplement: Supplementary file 2 — High resolution image (TIF 487 kb) [file 11095_2020_2816_MOESM1_ESM.tif]

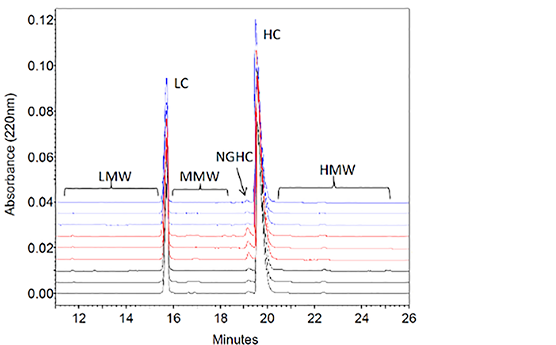

Supplement: Supplementary file 3 — (PNG 565 kb) [file 11095_2020_2816_Fig9_ESM.png]

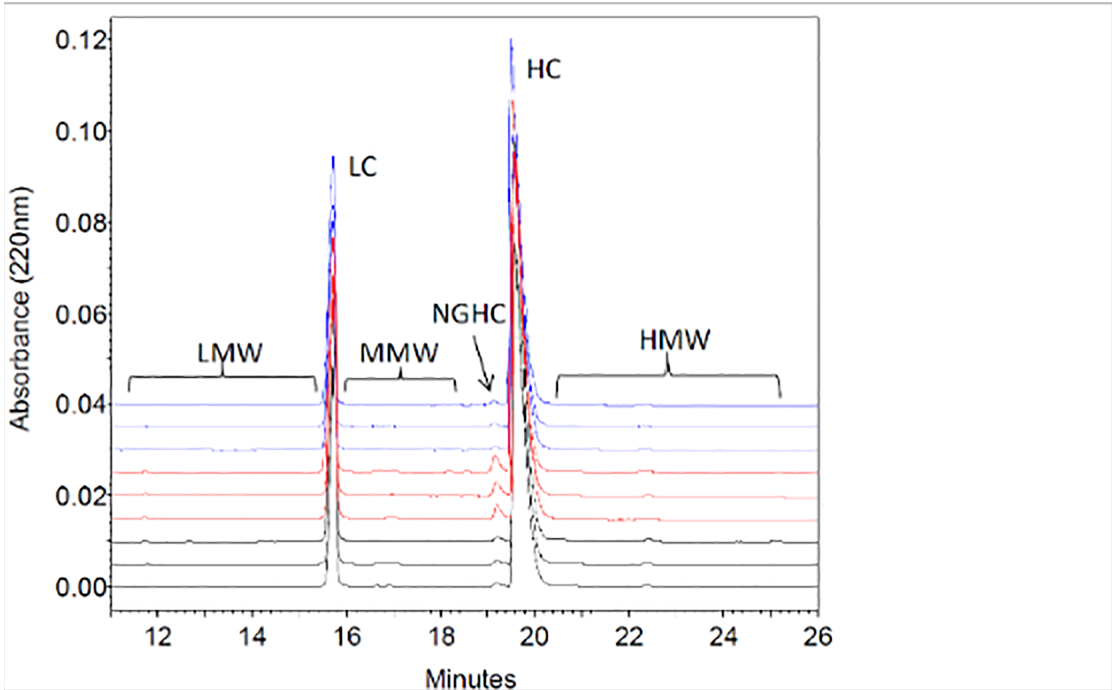

Supplement: Supplementary file 4 — High resolution image (TIF 580 kb) [file 11095_2020_2816_MOESM2_ESM.tif]

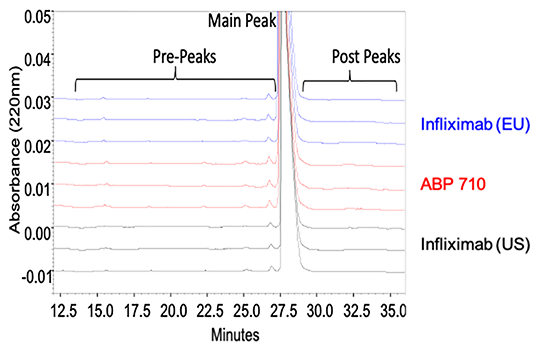

Supplement: Supplementary file 5 — (PNG 563 kb) [file 11095_2020_2816_Fig10_ESM.png]

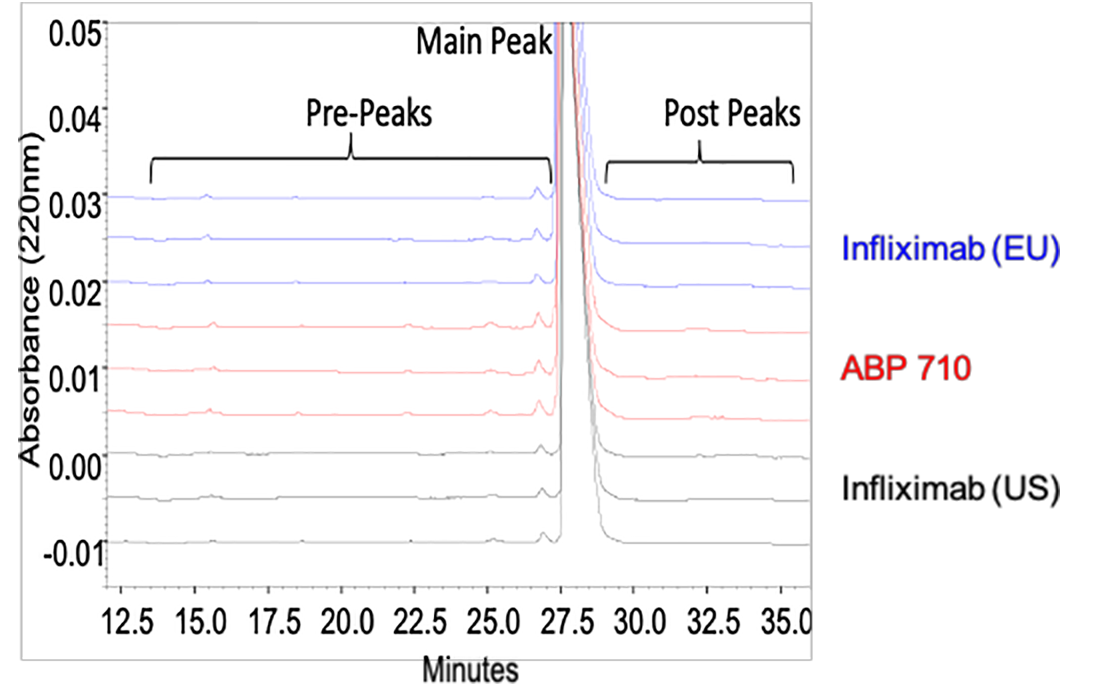

Supplement: Supplementary file 6 — High resolution image (TIF 615 kb) [file 11095_2020_2816_MOESM3_ESM.tif]
