# Supplementary material for: Analytical and Functional Similarity Assessment of ABP 710, a Biosimilar to Infliximab Reference Product
Source: Pharm Res. 2020 May 31;37(6):114. doi: 10.1007/s11095-020-02816-w (PMC7261735; doi:10.1007/s11095-020-02816-w)
Supplement: Supplementary file 7 — (DOCX 15 kb) [file 11095_2020_2816_MOESM4_ESM.docx]

**Online Resource 1**

A comparison of the SE‑UHPLC results for ABP 710, infliximab (US), and infliximab (EU)

**Online Resource 2**

A comparison of rCE‑SDS results for ABP 710, infliximab (US) and infliximab (EU)

**Online Resource 3**

A comparison of nrCE‑SDS results for ABP 710, infliximab (US) and infliximab (EU)
